# Long-term Exposure to Neighborhood Deprivation and Intimate Partner Violence Among Women: A UK Birth Cohort Study

**DOI:** 10.1097/EDE.0000000000001144

**Published:** 2020-02-03

**Authors:** Alexa R. Yakubovich, Jon Heron, Gene Feder, Abigail Fraser, David K. Humphreys

**Affiliations:** From the a Department of Social Policy and Intervention, University of Oxford, Oxford, United Kingdom; b Centre for Urban Health Solutions, Li Ka Shing Knowledge Institute, St. Michael’s Hospital, Unity Health Toronto, Toronto, Canada; c Department of Population Health Sciences, Bristol Medical School, University of Bristol, Bristol, United Kingdom; d MRC Integrative Epidemiology Unit at the University of Bristol, Bristol, United Kingdom; e Centre for Academic Primary Care, Bristol Medical School, University of Bristol, Bristol, United Kingdom.

**Keywords:** Intimate partner violence, Longitudinal studies, Neighborhood, United Kingdom, Women

## Abstract

Intimate partner violence is the most common form of violence perpetrated against women. To our knowledge, the effect of neighborhood disadvantage on intimate partner violence against women has never been investigated prospectively outside the United States.

**Methods:**

We used data from the Avon Longitudinal Study of Parents and Children in the United Kingdom, which followed our target sample, 7,219 women, from birth and their mothers (from pregnancy). At age 21, 2,128 participants self-reported the frequency of experiencing physical, psychological, or sexual intimate partner violence since age 18. Participants’ exposure to neighborhood-level deprivation and family-level socioeconomic characteristics (e.g., income) were measured at 10 time points from baseline (gestation) until children were 18 years old. We estimated the effect of cumulative exposure to greater neighborhood-level deprivation on the risk of experiencing intimate partner violence using marginal structural models with stabilized inverse probability weights, accounting for time-varying confounding by socioeconomic indicators and sample attrition.

**Results:**

A one-unit increase in cumulative exposure to more severe neighborhood deprivation was associated with a 62% increase in participants’ frequency of experiencing intimate partner violence (95% confidence interval 11%, 237%) and 36% increase in their risk of experiencing any intimate partner violence (95% confidence interval 1%, 85%).

**Conclusions:**

In our study, cumulative exposure to greater neighborhood deprivation over the first 18 years of life was associated with women’s increased risk of experiencing intimate partner violence in early adulthood. Future studies should test this association across contexts, including underlying mechanisms, and evaluate preventive strategies that target structural disparities.

## Abstract 2

Supplemental Digital Content is available in the text.

One-third of women worldwide are estimated to have experienced physical and/or sexual violence from a current or former partner.^1^ This violence, along with psychological abuse, is known as intimate partner violence (IPV) and is the most common form of violence perpetrated against women, with consequences including death, injury, and psychological disorders.^2–4^ Cross-sectional studies, including innovative spatial analyses,^5–8^ have largely established a positive association between neighborhood disadvantage and IPV against women.^9–12^ In contrast, there has been a relative dearth of longitudinal studies of this association, showing mixed findings,^13^ which limits causal conclusions and implications for preventive interventions.

Typically hypothesized mechanisms for the relationship between neighborhood deprivation and IPV are grounded in social disorganization theory^14^ or, its extension, collective efficacy theory.^15^ These theories postulate that in neighborhoods facing greater socioeconomic disadvantage and residential instability, it is more difficult to establish the social ties and informal social control that minimize violence and maximize intervention capacity. While developed to explain neighborhood-level variations in *public* criminality (e.g., vandalism, burglary),^15–18^ these theories have been extended to *private* forms of violence like IPV: for instance, with stronger neighborhood social ties and support structures expected to “steer women away” from violent partners, provide resources for leaving violent relationships, and create a more inhibitory environment (e.g., where neighborhood members are perceived as able and willing to regulate private violent behavior).^19^ Of relevance to a developmental context, sustained exposure to neighborhood disadvantage has further been hypothesized to increase the risk of IPV by normalizing aggression, for instance, via higher rates of neighborhood violence.^9–12^ Likewise, this exposure is expected to increase trauma or stress and thus vulnerability to future victimization by exacerbating individual-level risks such as substance misuse or social isolation, increasing relational strain, or inhibiting help-seeking behaviors.^9–12,20^

In contrast, a meta-analysis of three available and combinable longitudinal studies of neighborhood disadvantage and IPV against women found a small but protective association, underscoring the need for further investigation.^13^ To our knowledge, there are two additional prospective studies (see eTable 1; http://links.lww.com/EDE/B620, eAppendix 1; http://links.lww.com/EDE/B620).^21–25^ Four of five of these studies were US based, and all adjusted for postexposure or cross-sectional covariates that may be on the causal pathway (e.g., socioeconomic status), potentially underestimating the total effect of neighborhood disadvantage or, at worst, inducing collider stratification bias.^26–28^ Additionally, four studies did not account for duration of exposure.^28–31^ The fifth study measured exposure and outcome over two contemporaneous time points, limiting causal conclusions.^21^

We aimed to build on this evidence base by investigating, for the first time, the effect of long-term exposure to neighborhood-level deprivation on the risk of experiencing IPV among women using 21 years of prospective longitudinal data in the United Kingdom. Socioeconomic and psychosocial characteristics of the family environment affect the neighborhoods that families live in and are in turn affected by neighborhood environments; these family characteristics may additionally affect the risk of experiencing IPV in early adulthood (see eFigure 1; http://links.lww.com/EDE/B620, eAppendix 1; http://links.lww.com/EDE/B620).^13,28,32^ As a result, adjusting for socioeconomic indicators, as in prior studies, may partial out part of the effect of long-term neighborhood deprivation on IPV and induce collider stratification bias, whereas not controlling for these indicators would result in confounding. We therefore used marginal structural models, where we first weighted each participant by the inverse of the probability of experiencing their observed neighborhood exposure at each time point given their prior exposure and covariate history.^27,33,34^ This creates a pseudo-population, where the probability of exposure is comparable across covariate levels at each time point. Analyses of the exposure–outcome association within this weighted sample account for confounding by covariates measured before each exposure, without removing the indirect effects of exposure to neighborhood deprivation via these covariates at later times (see eAppendix 1; http://links.lww.com/EDE/B620 for further details).

## METHODS

Data are from the Avon Longitudinal Study of Parents and Children (ALSPAC), an ongoing birth cohort study. All pregnant women resident in three health districts in Avon, UK (including urban and rural areas) due between 1 April 1991 and 31 December 1992 were eligible to participate.^35,36^ Initially, 14,541 pregnancies were enrolled.^13^ When children were age 7, eligible mothers not enrolled were contacted, increasing the sample to 15,454 pregnancies (14,901 babies alive at age 1, 76% of all eligible)— these children comprise the ALSPAC birth cohort. Our target sample was the 7,219 girls enrolled. The ALSPAC Ethics and Law Committee and local research ethics committees provided ethical approval. Informed consent was obtained from participants after the recommendations of the ALSPAC Ethics and Law Committee at the time.

### Measures

#### IPV

Of the 7,219 women with baseline data, 2,128 responded at age 21 to a previously validated eight-item scale on physical, psychological, and sexual IPV experiences (from 0 = never to 3 = often) before and/or after age 18 (Table 1, *α* = 0.95).^37^ To delineate temporality, we only analyzed IPV between ages 18 and 21. Participants who experienced any IPV also self-reported on experiencing any of eight negative impacts from this violence: scared, upset, work/studies affected, sad, anxious, increased alcohol or substance use, angry, or depressed. The measure was developed by a team of IPV researchers based on questionnaires used with young people^38,39^ and a clinical sample^40^ in Bristol and piloted for acceptability with the ALSPAC participant advisory group.

**TABLE 1.**
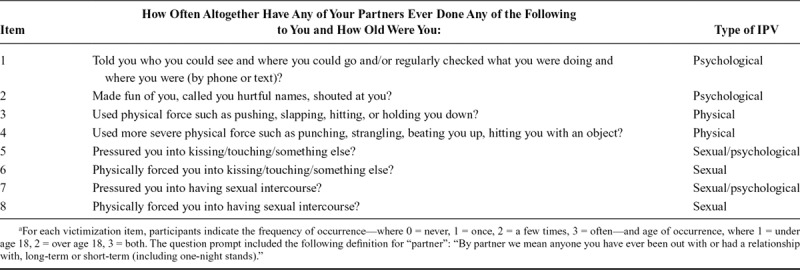
IPV Items

We analyzed two primary outcomes: (1) participants’ sum scores across the eight IPV items (0–24), which reflected the average frequency or intensity across all IPV experiences and (2) any IPV experience between ages 18 and 21.

#### Neighborhood-level Deprivation

We measured exposure to neighborhood-level deprivation using the 2010 Indices of Multiple Deprivation.^41^ This is an official measure of area-level deprivation in England, which considers deprivation beyond economic poverty alone, using indicators across seven domains: income, employment, education, health, crime, housing, and living environment (eTable 2; http://links.lww.com/EDE/B620, eAppendix 2; http://links.lww.com/EDE/B620).^42,43^ Based on these indicators, each neighborhood in England—specifically, lower layer super output area (~1,500 residents or 650 households designed to approximate residential neighborhoods)—is assigned a domain-specific and total deprivation rank score relative to all other neighborhoods (see eAppendix 2; http://links.lww.com/EDE/B620 for further discussion).

In the current study, we had access only to participants’ quintile ranks for each domain and the total deprivation index—determined by ALSPAC to protect anonymity—so that at each time point (see Table 2), we knew the quintile of deprivation each participant’s residential neighborhood fell into relative to all other neighborhoods in England (i.e., not just within ALSPAC). Owing to the underlying exponential distribution of the rank scores (as shown in Figure), differences in deprivation are greater between the most deprived quintiles compared with the least and there is less differentiation in deprivation between the less deprived quintiles.^43^ We therefore did not expect to find a linear trend in IPV risk among the quintiles nor for this to be the most meaningful contrast of neighborhood deprivation. Ideally, we would have analyzed participants in quintile 5 (i.e., the 20% most deprived neighborhoods in England) versus all others; however, we lacked the appropriate statistical power, with a low proportion of ALSPAC participants in quintile 5 alone dropping to <6% at later time points. We thus used a binary contrast for each time point where 1 = quintiles 4 and 5 (participants living in the top 40% most deprived neighborhoods in England) and 0 = otherwise. We expected this to be a more conservative test of the effect of neighborhood deprivation.

**TABLE 2.**
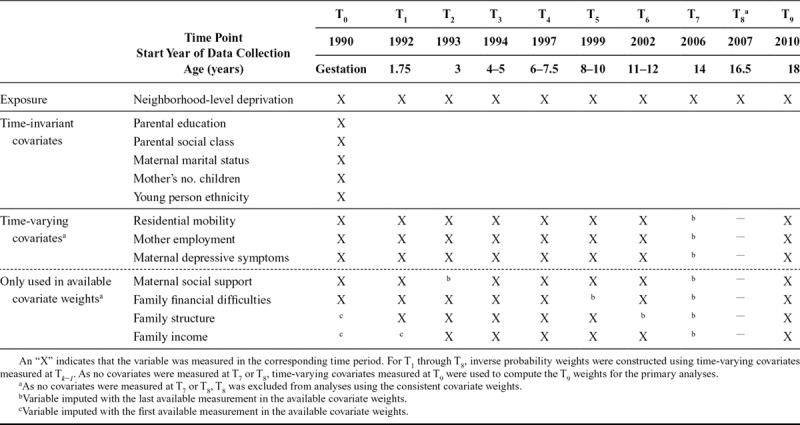
Data Availability for Exposure and Covariates Used in Inverse Probability of Exposure and Censoring Weights

**FIGURE.**
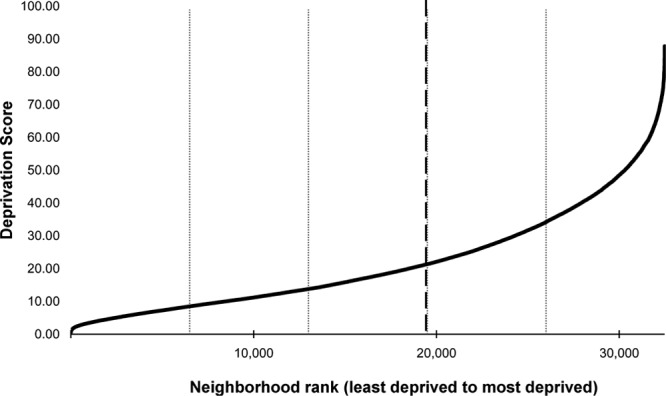
Exponential distribution of 2010 Indices of Multiple Deprivation scores by neighborhood (lower layer super output area) rank. The fine dashed lines indicate quintile markers. Neighborhoods to the right of the thick dashed line are the 40% most deprived neighborhoods in England. Data are from the official English Indices of Multiple Deprivation 2010 (available at https://www.gov.uk/government/statistics/english-indices-of-deprivation-2010); further details on the measure and its construction are in the English Indices of Multiple Deprivation 2010 technical report.^41^

We used inverse probability weights that predicted the probability of participants’ neighborhood deprivation exposure at each time point until age 18. To estimate the causal effect of neighborhood-level deprivation on IPV—given 2^10^ (1,024) possible exposure trajectories—a parametric model was necessary.^27^ Based on similar longitudinal studies,^28,30,44^ we used participants’ duration weighted exposure, where we computed the mean of each participant’s level of deprivation exposure (0,1) across the time points analyzed to compare participants who lived in more versus less deprived neighborhoods for longer. This specification was of theoretical interest because it allowed for estimation of the effects of *sustained* exposure to neighborhood deprivation (i.e., differences in IPV risk between women who spent more of their childhood in the most deprived neighborhoods in England versus the least).

#### Covariates

The following variables were only available at baseline and included as *time-invariant covariates*: parental education, maternal marital status, parental occupational social class, mother’s number of children, and the young person’s race/ethnicity. The following *time-varying covariates* were measured throughout the study: maternal depressive symptoms, maternal social support, residential mobility, parental employment status, family structure, family financial difficulties, and family income. Covariates were selected based on our directed acyclic graph—included, along with full details on covariate measures, in eAppendix 2; http://links.lww.com/EDE/B620. Further details are available in a searchable data dictionary at http://www.bristol.ac.uk/alspac/researchers/our-data/.

#### Analysis

We used marginal structural models with inverse probability weights, which provide an unbiased estimate of the causal effect of neighborhood deprivation on IPV under the following assumptions: exchangeability (no unmeasured confounding); consistency (unambiguously defined exposure); positivity (nonzero probability of each possible exposure value at each possible confounder value); and correct model specification of the marginal structural model and weights.^45^ We conducted analyses in Stata 13.1 (StataCorp LP, College Station, TX). Full details on estimating the weights and marginal structural models (including code and equations) are in eAppendix 2; http://links.lww.com/EDE/B620.

#### Exposure Model

As ALSPAC often consisted of multiple assessments per year measuring different variables, we combined assessments to create 11 time periods: 10 time periods from baseline (gestation) until age 18 where variations in exposure and confounders were measured and the age 21 outcome. There were two ways to compute the exposure weights: using only those confounding variables measured in all 10 time periods (consistent covariate weights) or using all available confounders (available covariate weights). We ran analyses twice using each set of weights to assess robustness. For the available covariate weights, when variables were not measured in a given period, we used the most recent measurement (last observation carried forward or, for family structure and income, first observation carried backward). Table 2 summarizes the variables used in each set of weights.

We used stabilized weights for more efficient estimates.^33^ To estimate the denominator of the weights, we regressed level of neighborhood deprivation at time *k* (T_*k*_) onto previous exposure and time-varying covariates at T_*k−1*_ and baseline covariates using binary logistic regression. The only exception was for the T_9_ weights, which used time-varying covariates measured at T_9_ as none were measured at T_8_. We estimated the numerator in the same way as the denominator but excluding the time-varying covariates. We checked the distribution of these stabilized weights for extreme values or high variability, which suggest violations of the assumptions of positivity and correct model specification.^45^ To further confirm positivity, we checked that there were participants in each deprivation quintile at each level of the categorical confounders.

#### Missing Data

As in most long-term studies, ALSPAC experienced significant attrition (see eFigure 2; http://links.lww.com/EDE/B620, eAppendix 2; http://links.lww.com/EDE/B620). We accounted for missing data in several ways. First, in analyses of participants’ duration weighted exposure, participants had to have exposure data for at least 50% of the time points analyzed. Second, in our main analyses, in addition to the exposure weights, we computed stabilized weights for being permanently lost to follow up, or censored. The only difference from the exposure weights was that the probability of being censored (rather than neighborhood exposure) was predicted. These weights allowed for an effect estimate independent of nonrandom attrition conditional on observed data. We explored additional missing data strategies in sensitivity analyses as described below.

#### Marginal Structural Model

The final weights were the product of the exposure and censoring weights at each time point. The marginal structural model was either a negative binomial or log-binomial model where we modeled the IPV frequency score or risk of experiencing any IPV, respectively, as a function of cumulative exposure to neighborhood deprivation and baseline covariates in the weighted sample. Negative binomial regression allowed for estimation of incidence rate ratios, and consideration of the varying intensity of IPV, given the right-skewed, discrete distribution of the IPV frequency scores and overdispersion.^46^ The log-binomial model allowed for estimation of risk ratios.^47^ We conducted our analyses in a long format dataset, where each participant observation was weighted by the appropriate time-varying weight (i.e., cumulative probability of observed exposure and censoring history until time *t*) with cluster-robust (conservative) standard errors.^48^ This maximized our use of all available data, allowing us to include participants with at least 50% exposure data, covariate data, and complete outcome data (see eAppendix 2; http://links.lww.com/EDE/B620 for further discussion). We explored alternative strategies in our sensitivity analyses.

We addressed the (untestable) assumption of exchangeability by using a robust set of confounders and both possible weight formulations. To address correct model specification, we ran several sensitivity analyses.

#### Sensitivity Analyses

We ran three types of sensitivity analyses (n = 38 analyses), testing: (1) stricter IPV operationalizations (e.g., any IPV with a self-reported negative impact^37^), (2) additional missing data strategies (e.g., including weights for intermittent missingness^49^), and (3) alternative model specifications (e.g., using time-varying, point-in-time exposure^44^). Results did not meaningfully differ from our main analyses; sensitivity analyses are thus presented in full in eAppendix 3; http://links.lww.com/EDE/B620. We also ran crude (adjusting for all time-invariant and time-varying covariates at baseline) and adjusted analyses (additionally adjusting for average time-varying covariates over the remaining study period) in an unweighted sample (i.e., not accounting for time-varying confounding) for more conventional estimates.

#### Secondary Analyses

We explored alternative hypotheses in secondary analyses. First, we tested whether economic deprivation alone (income deprivation) or higher neighborhood-level crime (crime deprivation) was associated with IPV. Second, we tested our assumption regarding the nonlinear trend of IPV risk across the deprivation quintiles by rerunning analyses using this ordinal variable as the exposure. Third, as prior validation of the IPV scale showed evidence for either a single factor or two factors of physical/psychological and sexual IPV, we reran analyses with these as separate outcomes.^37^ Finally, extending the only prior longitudinal study of IPV against women to analyze change in neighborhood disadvantage exposure,^21^ we examined whether most recent or past exposure to more deprived neighborhoods was most important to IPV compared with no exposure.

## RESULTS

Most participants were White, had parent(s) with higher-level educations or occupations, or had a married mother at baseline (Table 3). As expected, the proportion of participants whose parents lived with them, were employed, or had recently moved decreased over time. Family financial difficulties and maternal social networks improved and maternal depressive symptoms worsened slightly over time.

**TABLE 3.**
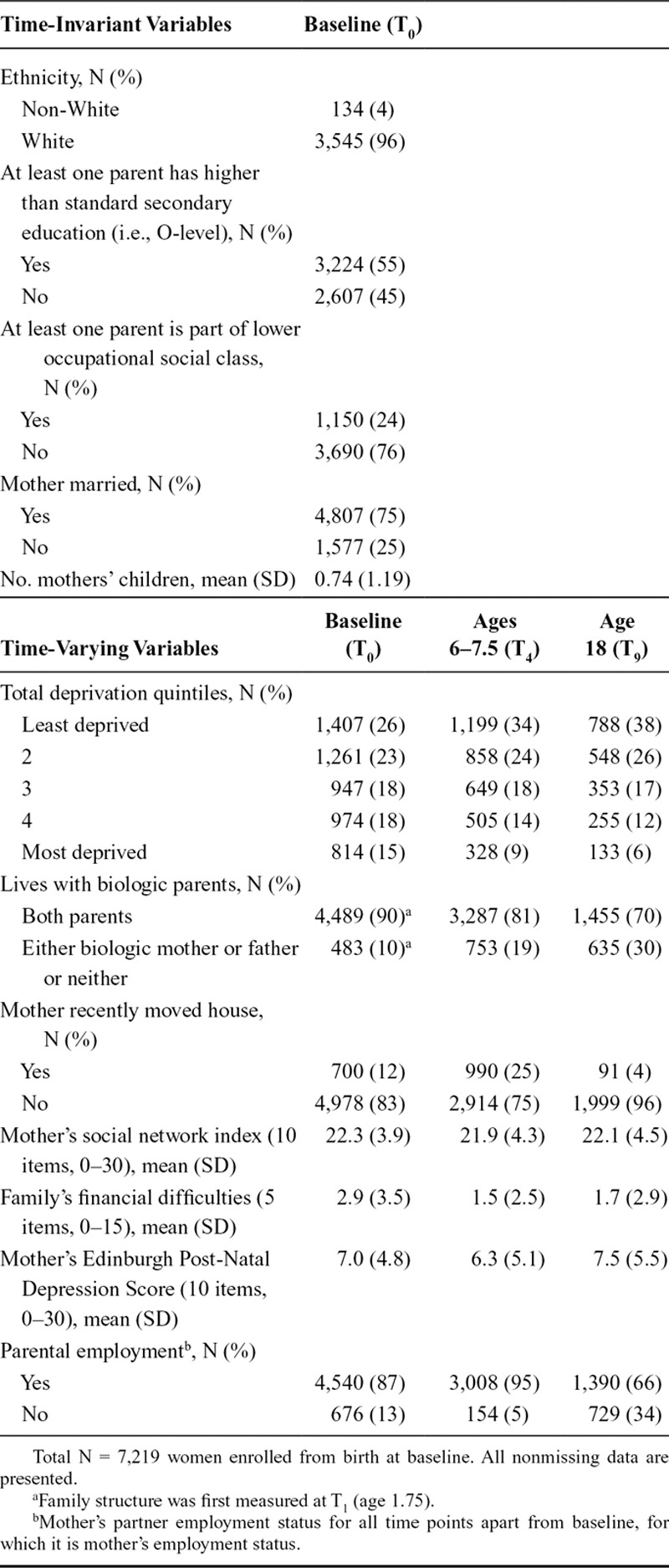
Sample Characteristics

At baseline, 33% of participants lived in the most deprived neighborhoods (quintiles 4 and 5), whereas 67% lived in the least (quintiles 1–3). Over time, the proportion of participants living in the most deprived neighborhoods decreased, influenced partly by nonrandom attrition (eAppendix 3; http://links.lww.com/EDE/B620, eFigure 3; http://links.lww.com/EDE/B620). eFigures 4–6; http://links.lww.com/EDE/B620 (eAppendix 3; http://links.lww.com/EDE/B620) demonstrate the within-participant mobility and variation over time in exposure to neighborhood deprivation. For instance, 54% of participants experienced a change in deprivation quintile during the study period (eFigure 5; http://links.lww.com/EDE/B620).

Between ages 18 and 21, 32% of women experienced any IPV (n = 683); 29% experienced any IPV with at least one negative impact (n = 608). The average IPV frequency score (range: 0–24) was 1.48 [standard deviation (SD) = 3.08]. Among those who had experienced any IPV (n = 683), the mean frequency score across all items was 4.61 (SD = 3.90). eTable 3; http://links.lww.com/EDE/B620 (eAppendix 3; http://links.lww.com/EDE/B620) provides the prevalence estimates for each IPV item and self-reported negative impact.

### Main Analyses

All formulations of the weights were stable (M ≈ 1.0, SD < 1.0) (Table 4). The distribution of deprivation quintile scores by categorical confounders further indicated the assumption of positivity was satisfied (eAppendix 3; http://links.lww.com/EDE/B620, eTables 4 and 5; http://links.lww.com/EDE/B620).

**TABLE 4.**
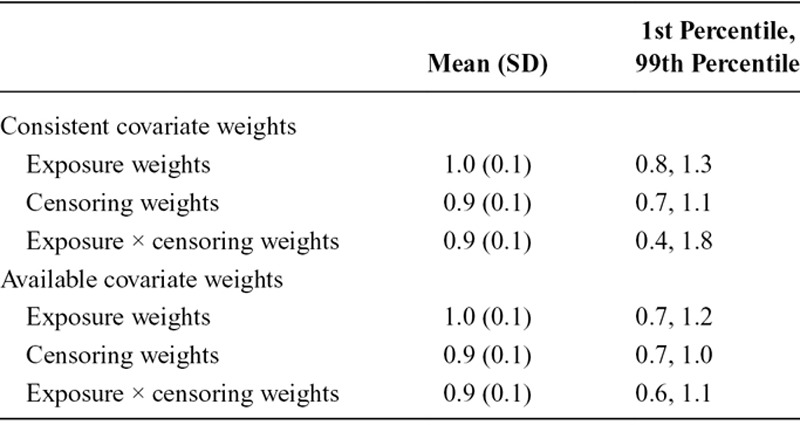
Mean, SD, and Range of Stabilized Exposure and Censoring Weights for Main Analyses

Table 5 shows the effect estimates for cumulative exposure to neighborhood deprivation on women’s risk of experiencing IPV. Based on the most conservative estimates, a 1-unit increase in cumulative exposure to the most versus the least deprived neighborhoods over the study period was associated with an average 62% increase in women’s IPV frequency scores [95% confidence interval (CI) 11%, 237%] and 36% increase in their risk of experiencing any IPV (95% CI 1%, 85%) (using the consistent covariate and available covariate weights, respectively).

**TABLE 5.**
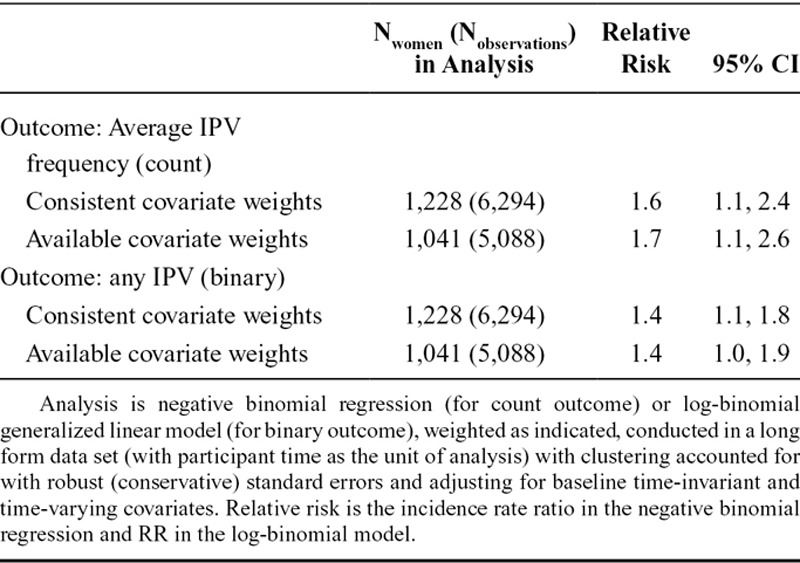
Effect Estimates of Exposure to More Severe Neighborhood Deprivation on IPV Among Women from Marginal Structural Models

### Sensitivity Analyses

Diagnostics and results for sensitivity analyses were similar to our main analyses and are given in eTables 6 and 7; http://links.lww.com/EDE/B620 (eAppendix 3; http://links.lww.com/EDE/B620). The smallest estimate observed for cumulative neighborhood deprivation was for the effect on any IPV with a negative impact accounting for the consistent covariates [risk ratio (RR) = 1.3, 95% CI 1.0, 1.7]. Estimates of the average effect of point-in-time exposure on IPV risk were also smaller but positive (e.g., any IPV: RR = 1.2, 95% CI 1.0, 1.6, using available covariates). Results from our adjusted models in the unweighted sample were further consistent.

### Secondary Analyses

Secondary results are given in eTable 8; http://links.lww.com/EDE/B620 (eAppendix 3; http://links.lww.com/EDE/B620). First, neither crime nor (especially) income deprivation showed consistent, clinically meaningful associations with IPV when analyzed separately, with 95% CIs tending to center around the null. Second, as expected, the association between deprivation quintiles and IPV was positive but weak regardless of the weights or outcome analyzed. Third, cumulative exposure to neighborhood deprivation was positively associated with more frequent, and any experience of, physical/psychological and sexual IPV. Point estimates (ranging from 1.3 to 2.3) and CIs tended to be larger for sexual IPV, possibly owing to its lower prevalence and collapsing of heterogeneous experiences (e.g., those who experienced physical or psychological but not sexual IPV were classified as 0). Finally, we compared participants (1) living in a deprived neighborhood at age 18 or (2) not living in a deprived neighborhood at age 18 but with prior exposure to (3) those who had never lived in more deprived neighborhoods. Those with prior exposure to more deprived neighborhoods but whose neighborhoods at age 18 were less deprived were at highest risk of experiencing IPV (RR = 1.6, 95% CI 1.1, 2.1).

## DISCUSSION

To our knowledge, this is the first prospective investigation of the effect of long-term exposure to neighborhood-level deprivation on IPV against women. Long-term exposure to more compared with less deprived neighborhoods over the first 18 years of life was associated with more frequent IPV and 36% higher risk of any IPV in early adulthood across a variety of models. Prior prospective longitudinal studies (mostly US-based) of the association between neighborhood disadvantage and IPV against women have shown mixed findings.^21–25^ Our findings may have been partly driven by uniquely considering long-term variation in neighborhood deprivation exposure over a significant developmental period. This variation—whether a person spends 1 year in a more deprived neighborhood versus several years or a decade—is important when considering the effects of neighborhood deprivation, especially in the context of potential developmental mechanisms.^29,50,51^ For instance, longer exposure to greater neighborhood-level deprivation over childhood has been shown to be associated with decreased cognitive ability^30^ and educational attainment^28^ and increased odds of early parenthood.^52^ Our findings extend this evidence base and suggest that cumulative exposure to more severe neighborhood deprivation over childhood also increases women’s eventual risk of experiencing IPV.

Our findings are consistent with the hypothesis of a small effect of long-term exposure to neighborhood deprivation on IPV, which is not unusual in the neighborhood or developmental effects literature, likely because these factors are more distal or upstream to the outcome.^28,44,53,54^ Nevertheless, understanding the effects of upstream factors over time—and how they interact with more proximal factors—is necessary for a rigorous etiologic understanding of IPV, and indeed most, if not all, complex public health problems.^55–57^ This is critical to designing interventions that can improve the well-being of populations rather than only individuals.^58,59^

Although qualitative studies have explored the perceived role of neighborhood deprivation in causing and exacerbating IPV (e.g., via psychological trauma or service/support barriers),^60–64^ longitudinal studies testing the mechanisms underlying this effect are still needed. The current study focused on a measure of relative deprivation: how long-term neighborhood inequalities affect women’s risk of experiencing IPV (e.g., by establishing normative standards or exacerbating psychosocial stress) and whether findings extend to absolute deprivation should be investigated. Moreover, we found that exposure to multiple deprivation, rather than singular dimensions of income poverty or higher crime, was the most influential in increasing women’s risk of experiencing IPV. This supports a conceptual framework where the accumulation of deprivation across material and social conditions is the key construct in defining neighborhood effects.^41,65,66^ The replicability of our findings under alternative definitions of multiple deprivation should be explored. Finally, our secondary analyses suggested that recently moving to a less deprived neighborhood after prolonged deprivation exposure was most harmful. Future research should formally investigate how different trajectories of neighborhood deprivation exposure affect IPV and possible critical or sensitive periods.^16^

Neighborhood effects research has historically focused on identifying *contextual* effects of neighborhoods over and above compositional effects (i.e., due solely to the characteristics of neighborhood residents).^67,68^ However, this convention disregards the role of compositional factors in the hypothesized causal mechanisms underlying contextual effects and may artificially reduce neighborhood effect estimates.^50,69^ In other words, contextual and compositional effects are not mutually exclusive but rather “inextricably linked.”^70^ In using marginal structural models, we sought to account for the individual socioeconomic and psychosocial factors that predict neighborhood selection, while avoiding adjustment for later values of these variables, hypothesized as mediators of exposure. Differences between these and our conventionally adjusted estimates were small, as in prior comparisons^71^—we would expect greater differences in samples with greater variation in neighborhood exposure over time (e.g., owing to greater social mobility). Nevertheless, we hope to contribute to the continued practice of making causal assumptions explicit and avoiding overadjustment for individual-level factors.

This study has several limitations. First, our estimate is biased as a causal association if there is an unobserved variable that predicts both neighborhood deprivation exposure and IPV independent of our included covariates. Our findings were robust across several model specifications using a rich set of baseline and time-varying socioeconomic and psychosocial covariates, which reduces this concern. However, it is worth noting that the interaction between individual characteristics and neighborhood environments is a social process in itself.^16,72^ The existence of residual individual-level characteristics that predict neighborhood selection does not necessarily negate the importance of the neighborhood; rather these interactions should be further investigated as potential effects. Additionally, it is unlikely that reverse causality underlies our findings (i.e., that experiencing IPV caused participants to move to or stay in more deprived neighborhoods), given the timing of exposure considered (ages 0–18) and that for most participants (70%) the outcome measured was their first experiences of IPV.

Second, the Indices of Multiple Deprivation measure census-based, residential neighborhoods,^42^ which may not align with participants’ conceptualizations of their neighborhoods or daily activities, and different definitions of neighborhoods can drastically alter results (the modifiable areal unit problem).^73–75^ Future research should consider work, school, and social environments and triangulate across measures. Third, we used one iteration of the deprivation scores rather than multiple iterations over time. This ensured that changes in quintiles indicated changes in participants’ deprivation exposure rather than measurement differences, but it means that we could not analyze changes in neighborhoods over time. Nonetheless, we do not expect this to bias our results, given that change in relative neighborhood deprivation in the study area (especially across quintile boundaries) was fairly minimal over the study period.^76,77^

Fourth, without access to the continuous scores, the exponential distribution of the Indices of Multiple Deprivation necessitated dichotomizing our exposure. We were further underpowered to test a stricter dichotomization of neighborhood deprivation. Nevertheless, dichotomized measures of neighborhood deprivation are common in the literature and central to hypotheses of threshold effects;^9,16^ given the more liberal threshold we examined, we expect our results are conservative, which should be explored using alternative operationalizations in future studies. Our sensitivity analyses showed that our results were at least robust to whether we estimated the effect of cumulative deprivation exposure (continuous and constant within participants) or the average effect of living in more versus less deprived neighborhoods at each time point (time-varying and binary).

Fifth, we did not have data on women’s partners nor the perpetrators and timing of IPV incidents. Finally, persons who were White and from higher socioeconomic families were overrepresented compared with the national population, which may add to the potential conservatism of our results. Future research should test generalizability and effect modification, including whether effects persist beyond early adulthood.

In conclusion, this study used rich data from a birth cohort sample with substantial variation in neighborhood deprivation exposures, a clinically meaningful measure of IPV, and robust family-level confounders over time. Our novel application of marginal structural models allowed us to estimate the potential causal effect of long-term exposure to neighborhood deprivation on IPV, conditionally independent of differential selection into neighborhoods and study attrition. Supporting the hypothesis of a sustained neighborhood effect on IPV among women over a 21-year period, our findings highlight the need for more longitudinal studies of contextual risk factors (and mechanisms) for IPV as well as the evaluation of preventive strategies that target and account for the structural determinants of these conditions.

## ACKNOWLEDGMENTS

The authors would like to acknowledge the contribution of Rhian Daniel who provided invaluable statistical guidance in the conduct of analyses and reporting of results and Michael Noble and Mark Fransham for their guidance on using the Indices of Multiple Deprivation. We would also like to acknowledge the work of Christine Barter, Marianne Hester, and Eszter Szilassy in developing the IPV scale used in ALSPAC, along with Gene Feder. We are extremely grateful to all the families who took part in ALSPAC, the midwives for their help in recruiting them, and the whole ALSPAC team, which includes interviewers, computer and laboratory technicians, clerical workers, research scientists, volunteers, managers, receptionists, and nurses.

## Supplementary Material


