# Supplementary material for: Long-term Exposure to Neighborhood Deprivation and Intimate Partner Violence Among Women: A UK Birth Cohort Study
Source: Epidemiology. 2020 Feb 3;31(2):272–81. doi: 10.1097/EDE.0000000000001144 (PMC7004477; doi:10.1097/EDE.0000000000001144)
Supplement: Supplementary file 1 [file ede-31-272-s001.pdf]

eAppendix 1: Background

***eTable 1:* Summary of prior prospective-longitudinal studies of association between neighbourhood disadvantage and IPV against women**

		Neighbourhood disadvantage exposure			Covariates adjusted for that may be on causal pathway			
**Authors (Cohort)**	**Country**	**Measure or construct (source)**	**N time points**	**IPV measure**	**Post or cross-sectional with exposure**	**Post or cross-sectional with exposure and outcome**	**Other relevant selection criteria**	**Association (analysis)**
Benson et al.[1,a] (National Survey of Families and Households)	USA	Top 25% most disadvantaged neighbourhoods measured by concentrated disadvantage index (census): 0=advantaged area both waves; 1=disadvantaged wave 1, advantaged wave 2; 2=disadvantaged both waves	2	Binary: Any physical IPV	-	Exposure is contemporaneous with outcome and all adjusted covariates, including:<br>- Employment instability<br>- Income-to-needs ratio<br>- Financial strain	Only participants who were cohabiting or married to the same partner in wave 2 as in wave 1: a common effect of living in less disadvantaged neighbourhoods and causes of IPV	B=0.31, SE=0.13 (binary logistic regression, exposure is ordinal variable but analysed continuously)
Giordano et al.[2,b] (Toledo Adolescents Relationship Study)	USA	Sum of neighbourhood problems (parent self-report)	1	Binary: Any IPV	-	Respondent and partner's:<br>- Delinquency<br>- Controlling behaviours<br>- Trait and relationship-based anger	-	OR=0.98, 95% CI 0.86, 1.10 (binary logistic regression)
Gomez et al.[3,b] (National Longitudinal Study of Adolescent Health)	USA	Concentrated disadvantage (census)	1	Ordinal: no, less severe, more severe physical or sexual IPV since age 18 over entire study period	-	- Retrospective child abuse<br>- Parental income<br>- Educational attainment	-	OR=0.91, 95% CI 0.84, 0.99 (ordered logistic regression)
Jain et al.[4,b] (Project on Human Development in Chicago Neighborhoods)	USA	Concentrated disadvantage (census)	1	Continuous: Sum of frequency of physical IPV items	- Perceived community violence<br>- Collective efficacy	-	By outcome measurement, >50% of the sample had moved from original neighbourhoods	B=-0.02, SE=0.33 (multilevel linear regression)
Leddy et al.[5,c] (HPTN 068 trial cohort)	South Africa	Wealthier (less unemployment, higher SES) and more permanent residents (census)	2	Binary: any physical IPV	- Collective efficacy<br>- High school enrolment[d]<br>- Household assets[d]	-	-	RR=0.99, 95% CI 0.95, 1.03 (modified GEE poisson regression)

USA is United States of America. B is unstandardised regression coefficient. SE is standard error. OR is odds ratio. CI is confidence interval. SES is socioeconomic status. RR is risk ratio. GEE is generalised estimating equation. Studies were identified through a systematic review of longitudinal studies of all risk and protective factors for IPV victimisation among adult women (search completed June 2016),[6] automated alerts on relevant literature via GoogleScholar (since June 2016), and additional focused searches (March 2019). Prospective-longitudinal studies were defined as studies with at least two time-points of data where exposure was measured prior to outcome or an analysis of change was conducted – note: this included studies that only had one time point of exposure data and one time point of outcome data, as was the case for most of the above studies. Studies were only included if majority of women were at least 18 years old at time of outcome assessment.

[a]A series of studies were conducted using data from the same cohort but either using one time point of cross-sectional neighbourhood exposure and IPV outcome data[7-9] or using two time points of data but analysing any IPV at both time points as the outcome.[10] As the study by Benson et al. provided the strongest longitudinal design, only this study is summarised above. The association between concentrated disadvantage and IPV against women was positive in all of these studies.

[b]Studies included in previous meta-analysis of the longitudinal association between neighbourhood disadvantage and IPV against women.[6]
[c]Identified in updated searches (published in 2018).
[d]Unclear if adjusted covariate was measured contemporaneously/after exposure (as opposed to prior).

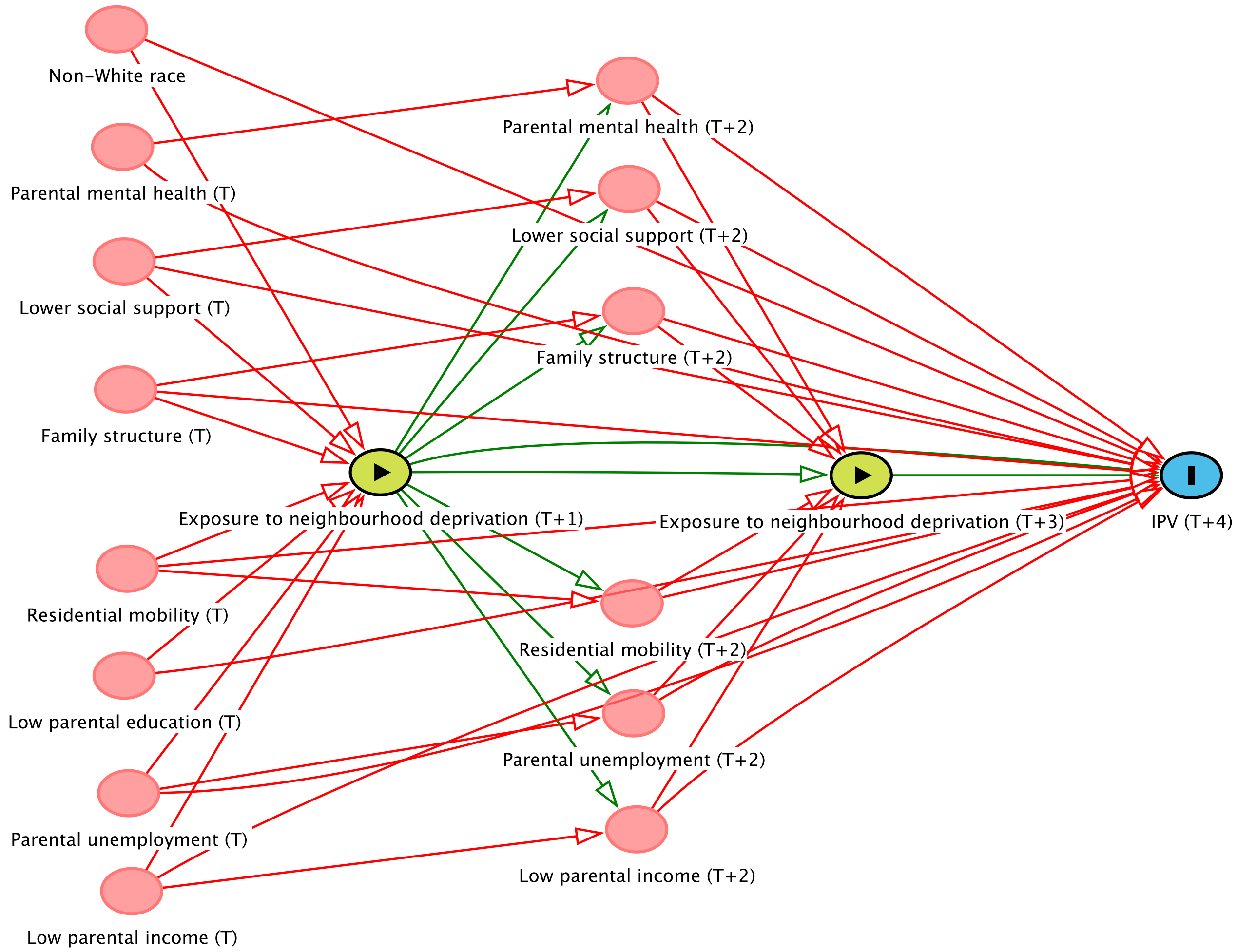

***eFigure 1:* Simplified directed acyclic graph (DAG) for the effect of exposure to neighbourhood disadvantage on IPV against women over time.** This DAG was drawn based on the most up-to-date synthesis of the longitudinal IPV literature[6] and, where gaps exist in this literature, hypotheses based on available cross-sectional evidence[11] and prior longitudinal studies of neighbourhood deprivation.[12-14] For simplicity, concurrent paths between confounders are not shown. Likewise, only variables that are hypothesised to confound the relationship between exposure to neighbourhood deprivation and IPV are included. We used DAGitty to create the figure.[15]

Marginal structural models estimated by inverse probability weighting

The current study sought to estimate the effect of exposure to neighbourhood deprivation from birth until age 18 on the risk of experiencing intimate partner violence (IPV) in early adulthood (ages 18-21). However, as depicted in the directed acyclic graph above (eFigure 1), socioeconomic and psychosocial characteristics of the family environment affect the neighbourhoods that families live in and are in turn affected by neighbourhood environments; these family characteristics may additionally affect the risk of experiencing IPV in early adulthood.[6,11,14] This is a classic case of time-varying confounding affected by past exposure, the simplest example of which is depicted below, where A is the exposure variable measured at T0 and T1, L is a vector of time-varying covariates at T0 and T1 and Y is the final study outcome:

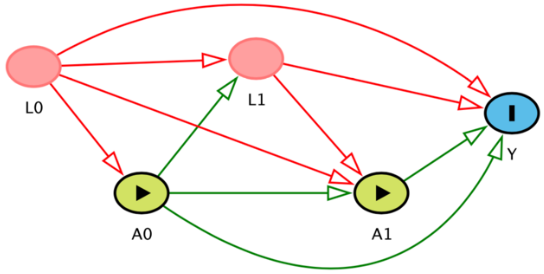

Formally, time-varying or time-dependent confounding affected by past exposure occurs when a time-varying exposure is both affected by the level of prior time-varying covariates and affects future values of those time-varying covariates – all of which then cause the outcome of interest.[16] The result is that, when estimating the effect of exposure on the outcome, controlling for the values of time-varying covariates using conventional regression methods would lead to 'over-adjustment' (partialling out part of the effect of the exposure on the outcome) and potentially induce collider-stratification bias, yet not controlling for these covariates would result in bias due to confounding.[17,18]

Marginal structural models allow for the estimation of a causal effect of a time-varying exposure in the presence of both time-varying confounders and mediators using observational data (under the assumptions of exchangeability, consistency, positivity, and correct model specification – as described in the main text).[18] Of note, these assumptions are the same as those required by conventional regression methods when estimating causal associations. In fact, the latter require the additional assumption that measured time-varying covariates are not affected by prior exposure – which is not required of marginal structural models.[14] The model is referred to as marginal because it estimates the average effect of exposure based on the marginal distribution of the counterfactual variables or potential outcomes (e.g., if the entire sample was exposed versus unexposed at time t) and structural because the estimate is of a causal effect.[19]

Marginal structural models can be estimated using inverse probability weighting. This essentially involves a two-step process, where (1) each participant's cumulative probability of experiencing the exposure history she actually experienced conditional on her covariate history and (2) running a crude analysis (i.e., without time-varying covariates) in a sample where each participant is weighted by the inverse of this probability. This means that participants whose exposure histories are more common given their covariate histories will be proportionally under-weighted in the analytic sample whereas participants whose exposure histories are less common given their covariate histories will be proportionally over-weighted. Intuitively, this creates a *pseudo-population*, where the probability of exposure is made comparable across levels of the confounders at each time point as though exposure had occurred at random (assuming no unmeasured confounders). As a result, marginal structural models estimated by inverse probability weighting can account for nonrandom selection of participants into neighbourhoods (e.g., due to socioeconomic variables) that may otherwise confound effect estimates but do not partial out the indirect effects of neighbourhood exposures via these variables at later time points.[14] They also are easy to interpret for researchers used to more conventional methods (e.g., generalized linear regression) as they are simply an extension of these models to weighted samples.[19] Of further benefit for use with cohort data, inverse probability weights can also be constructed for censoring or attrition, whereby, in addition to the exposure weights, participants in the analytic sample are also weighted by the inverse probability of remaining in the sample given their prior covariate and exposure histories – thus accounting for non-random attrition conditional on observed covariates. See '**Computing the stabilised inverse probability weights and estimating marginal structural models'** (eAppendix 2) for further details on the use of marginal structural models estimated by inverse probability weighting in practice and application in the current study.

eAppendix 2: Method

Measures

Indices of Multiple Deprivation

As described in the main manuscript, the Indices of Multiple Deprivation are an official measure of area-level deprivation in England, which considers deprivation beyond economic poverty alone, using indicators across seven domains: income, employment, education, health, crime, housing, and living environment. [20-22] eTable 2 describes the indicators used to construct each domain of deprivation and the domain's weight in the final index. Based on these indicators, each lower-layer super output area (~1500 residents or 650 households designed to approximate residential neighbourhoods) is assigned a domain-specific and total deprivation rank score relative to all other neighbourhoods. A neighbourhood is thus defined as more or less deprived based on the extent of inequality in its living conditions compared to other neighbourhoods in England (i.e., *relative* deprivation) as opposed to whether it falls above or below an objective standard of conditions (i.e., *absolute* deprivation). The Indices of Multiple Deprivation is constructed using an exponential transformation of neighbourhoods' rank scores; this standardises the scores for different indicators, allowing them to be combined, and makes the most deprived neighbourhoods easier to identify: the 10% most deprived neighbourhoods make up 50% of the score distribution (i.e., have a transformed-rank score between 50-100). See Figure 1 in the main manuscript for a summary of this distribution and the technical reports of the Indices of Multiple Deprivation for further details on the measure's construction and distribution.[20,22,23]

***eTable 2:* 2010 English Indices of Deprivation[20]**

Domain	Indicators	Weight in Index (2015)
Income deprivation	Adults and children in Income Support families;<br>Adults and children in income-based Jobseeker's Allowance families;<br>Adults and children in income-based Employment and Support Allowance families;<br>Adults and children in Pension Credit (Guarantee) families;<br>Adults and children in Child Tax Credit and Working Tax Credit families, below 60% median income not already counted;<br>Asylum seekers in England in receipt of subsistence support, accommodation support, or both.	22.5%
Employment deprivation	Claimants of Jobseeker's Allowance, aged 18-59/64;<br>Claimants of Employment and Support Allowance, aged 18-59/64;<br>Claimants of Incapacity Benefit, aged 18-59/64;<br>Claimants of Severe Disablement Allowance, aged 18-59/64;<br>Claimants of Carer's Allowance, aged 18-59/64.	22.5%
Education, skills, and training deprivation	Children and young people:<br>Key stage 2 attainment: average points score;<br>Key stage 4 attainment: average points score;<br>Secondary school absence;<br>Staying on in education post 16;<br>Entry to higher education.<br>Adults skills:<br>Adults with no or low qualifications, aged 25-59/64;<br>English language proficiency, aged 25-59/64.	13.5%
Health deprivation and disability	Years of potential life lost;<br>Comparative illness and disability ratio;<br>Acute morbidity;<br>Mood and anxiety disorders.	13.5%
Crime	Recorded crime rates for: Violence; Burglary; Theft; Criminal damage.	9.3%
Barriers to housing and services	Geographical barriers:<br>Road distance to: post office, primary school, general store/supermarket, GP surgery.<br>Wider barriers:<br>Household overcrowding;<br>Homelessness;<br>Housing affordability.	9.3%

Domain	Indicators	Weight in Index (2015)
Living environment deprivation	Indoor living environment:<br>Housing in poor condition;<br>Houses without central heating.<br>Outdoor living environment:<br>Air quality;<br>Road traffic accidents.	9.3%

Time-invariant covariates

All covariates were measured by mother-report, with time-invariant covariates measured at baseline, unless otherwise noted. Parental education was coded as 1=at least the mother or her partner had higher than O-level (A-level or degree), 0=otherwise. Maternal marital status was coded as 1=married, 0=otherwise (including widowed, divorced, separated, or never married). Parental social class was coded using the standard occupational classification 2000, where 1=at least mother or partner were part of lower social class (partly or unskilled occupations), 0=otherwise (professional, managerial, or skilled occupations). Mothers reported on their own, their partners', and their parents' race/ethnicities at baseline and on the young person's race/ethnicity when the child was age 11.5. To use all available data, participants coded as 'non-white' at age 11.5 were coded as 'non-white' on the final ethnicity variable and, in addition, those who were missing at the age 11.5 assessment but whose mothers, fathers, grandmothers, or grandfathers were reported as 'non-white' were also coded as such. Race/ethnicity was dichotomised as 1=non-white, 0=white because of the lower proportion of non-white race/ethnicities (e.g., Asian, Black) in the sample. We also used the mother's number of children at baseline as a time-invariant covariate.

Time-varying covariates

Maternal depressive symptoms were measured using the Edinburgh Post-Natal Depression Scale, a 10-item scale which asks about positive and negative behaviours/emotions in the last seven days (e.g., 'I have been anxious or worried for no good reason').[24] Response categories were 0 'Never' to 3 'Often'. Items were all coded in the negative direction and summed so that higher scores indicate more depressive symptoms (α=.85). Residential mobility was measured based on mothers' reports of whether they had moved house between questionnaire assessments. Maternal social support was measured using a Social Network Index developed for ALSPAC, where mothers reported on 10 social situations (e.g., 'How many of your relatives or your partner's relatives do you see at least twice a year?'). Response categories were 0 'None' to 3 'More than 4' and items were summed so that higher scores indicated a stronger social network (α=.79). Parental employment was typically indicated by mother-report of whether her partner was currently employed, except at baseline, when mothers reported on whether they themselves were currently employed. Family structure was coded as 1=both biological parents live with child, 0=only one or neither biological parent lives with child. Mothers reported on their difficulty in affording each of five items – food, clothing, heating, accommodation, or items for their child(ren) – on a 4-point scale from 0=not difficult to 3=very difficult. Responses were summed to create a composite for financial difficulties. Mothers reported on the take-home family income as a weekly average, apart from when children were age 18 when the monthly average was reported. Response categories varied over time, as expected with inflation, from baseline (weekly average, 1=<£100 to 5=>£400) to age 18 (monthly average, 1=<£899 to 10=>£4000).

Computing the stabilised inverse probability weights and estimating marginal structural models

Constructing inverse probability weights involves estimating a denominator that is the product of the probabilities that each participant received the exposure she actually did at time t conditional on prior exposure up until time t-1, time-varying covariates up until time t or time t-1 (depending on the study), and baseline covariates (including time-invariant covariates).[16,18,25,26] In practice, inverse probability

weights tend to be very variable; thus stabilised weights are typically used for more efficient effect estimates.[16,18] Stabilised weights involve estimating a numerator as well, which is typically a function of prior exposure and baseline covariates (i.e., excluding time-varying covariate history beyond baseline). The analysis in the weighted sample (i.e., marginal structural model) is then conducted conditioning on the baseline covariates.

In the current study, to compute the denominator of the stabilised weights, we regressed the level of neighbourhood deprivation at time k (A_k) onto the level of previous exposure (A_{k-1}) and time-varying covariates (L_{k-1}) at time k-1 and baseline covariates (X and L_0) using a pooled binary logistic regression in a long-form dataset (i.e., with participant-observations as the unit of analysis).[18,26] The only exception was for the weights constructed for T_9, which used time-varying covariates measured at T_9 because none were measured at T_8. We then estimated the predicted probabilities of exposure from the corresponding regression and derived participants' probabilities of their *observed* exposure status. The denominator for the final exposure weight at each time t is then the product of probabilities up until time t (i.e., the estimated probability of participants' observed exposure histories up until time t conditional on prior exposure and covariate history up until time t-1). In a wide-form dataset, where the unit of analysis is participants, this final weight would equate to the product of probabilities over all time points. We computed the numerator in the same way as the denominator but excluding the time-varying covariates beyond baseline (L_{k-1}) from the regression. The final form for the stabilised weights for the *i*th participant was thus:

$$SW_i = \prod_{k=1}^{K} \frac{p(A_{ki} = \alpha_{ki} | A_{(k-1)i} = \bar{\alpha}_{(k-1)i}, L_{0i} = l_{0i}, X_i = x_i)}{p(A_{ki} = \alpha_{ki} | \bar{A}_{(k-1)i} = \bar{\alpha}_{(k-1)i}, \bar{L}_{(k-1)i} = \bar{l}_{(k-1)i}, X_i = x_i)}$$

We estimated censoring (i.e., permanent attrition) weights (and where relevant weights for intermittent missingness) in the same way as the exposure weights, but instead predicting the probability of being censored (or missing). The final weights were the product of the stabilised exposure and censoring weights (and, where relevant, intermittent missingness weights).

As in any longitudinal analysis of an exposure-outcome association, estimating marginal structural models further requires defining the exposure trajectories (and potential outcomes) of interest. In simple settings (e.g., two time points of binary exposure), marginal structural models can be estimated non-parametrically, where the expected outcomes are compared for each possible exposure trajectory (e.g., $E(Y_{0,0})$, $E(Y_{0,1})$, $E(Y_{1,0})$, and $E(Y_{1,1})$).[16,18] However, in more complex settings, for instance due to many more time points, parametric models are required. In the current study, with 10 time points of binary exposure data, there were 2^{10} or 1,024 potential exposure trajectories. We thus estimated parametric marginal structural models. Based on prior marginal structural model studies of neighbourhood disadvantage, we used a cumulative or duration-weighted exposure, defined as the average exposure over the study period ($\sum_{k=0}^{9} a_k /10$).[12,14] As noted by these studies and the broader neighbourhood effects literature, this specification is of theoretical interest because it allows for estimation of the effects of sustained exposure to neighbourhood deprivation: for instance, in the current study, differences in IPV risk between women who spent more of their childhood in the most deprived neighbourhoods in England versus the least.

We estimated two primary marginal structural models in our weighted sample. One was a negative binomial regression, where the discrete IPV frequency score (Y) was modelled as a function of duration-weighted exposure to more severe neighbourhood deprivation ($\bar{\alpha}$) and (time-invariant and time-varying) covariates measured at baseline (X and L_0):

$$log[(Y_{\bar{\alpha}i} = y_{\bar{\alpha}i}|X_i = x_i, L_{0i} = l_{0i})] = \theta_0 + \theta_1\left(\frac{\sum_{k=0}^{9} a_{ki}}{10}\right)$$

The second was a log-binomial model, where the risk of experiencing any IPV (Y) was modelled as a function of duration-weighted exposure to more severe neighbourhood deprivation ($\bar{\alpha}$) and time-invariant/baseline factors (X and L_0):

$$log[P(Y_{\bar{\alpha}i} = 1)|X_i = x_i, L_{0i} = l_{0i}] = \theta_0 + \theta_1\left(\frac{\sum_{k=0}^{9} a_{ki}}{10}\right)$$

Typically, when the outcome is an end-of-study outcome as opposed to repeated measures, researchers will conduct their marginal structural models in a wide-form dataset, where each participant is weighted by the product of the time-specific weights.[13,14,16,27,28] This is analogous to the practice used in repeated-measures marginal structural models, where researchers weight each participant-observation at time k by the product of the time-specific weights up until time k.[12,19,26,28] However, in a wide-form dataset, this means that participants without complete data at all time points are listwise deleted. In the current study, this would have resulted in an analytic sample of n<200 participants. Therefore, to make the most of the available data, we conducted our analyses at the time-point level (i.e., in a long-form dataset), where each participant-observation was weighted by the appropriate time-varying weight (i.e., the cumulative probability of observed exposure and censoring history until time t) with cluster-robust (conservative) standard errors. This allowed us to include participants who did not have complete exposure and covariate data at all time points, but did have at least 50% exposure data, IPV data, and time points with complete covariate data (see eFigure 2 for further details). Participant observations are resultantly included in the analysis up until the time point with missing data (i.e., when the time-specific weight is missing). We explore alternative strategies in our sensitivity analyses.

STATA syntax for basic marginal structural model analysis

/*Denominator: computing pooled logistic regression to predict total IMD using only variables measured at all time points */

```
set more off
logit imd_b i.imd_b1 i.moved1 i.ptnremploy1 epds1 epds0 i.moved0 i.mumemploy0 i.eduparents0
i.mummarried0 nochild0 i.ethnicity0 i.parentsc0
```

***Predicting probabilities from logit for the estimation subsample**

```
predict pimd if e(sample)
replace pimd=pimd*imd_b+(1-pimd)*(1-imd_b)
```

/*Creating cumulative probabilities (multiplying each probability by the one before it, except for the first probability), clustered by participant id */

```
sort id time
by id: replace pimd=pimd*pimd[_n-1] if _n!=1
```

/*Numerator: computing pooled logistic regression to predict total IMD using only variables measured at all time points */

```
set more off
logit imd_b i.imd_b1 epds0 i.moved0 i.mumemploy0 i.eduparents0 i.mummarried0 nochild0 i.ethnicity0
i.parentsc0

predict pimd0 if e(sample)
```

replace pimd0=pimd0*imd_b+(1-pimd0)*(1-imd_b)

sort id time
by id: replace pimd0=pimd0*pimd0[_n-1] if _n!=1

/* Calculating weights (stabilized and unstabilized)

***Non-stabilised**
gen ipt_w=1/pimd
summarize ipt_w, detail

***Stabilised**
gen ipt_sw=pimd0/pimd
summarize ipt_sw, detail

***Checking distribution of weights**
summarize ipt_w, detail
summarize ipt_sw, detail

/*Now creating censoring weights*/

/*Denominator: computing pooled binary logistic regression to predict censoring using only variables measured at all time points*/
set more off
logit censor i.imd_b1 1 i.moved1 i.ptnremploy1 epds1 epds0 i.moved0 i.mumemploy0 i.eduparents0 i.mummarried0 nochild0 i.ethnicity0 i.parentsc0

***Predicting probabilities for the estimation subsample**
predict pcens if e(sample)

***Calculating probability of being uncensored**
replace pcens=1-pcens

/*Calculating cumulative probabilities (multiplying each probability by the one before it, except for the first probability), clustered by participant id*/
sort id time
by id: replace pcens=pcens*pcens[_n-1] if _n!=1

/*Numerator: computing pooled binary logistic regression to predict censoring using only variables measured at all time points */
set more off
logit censor i.imd_b1 epds0 i.moved0 i.mumemploy0 ///
i.eduparents0 i.mummarried0 nochild0 i.ethnicity0 i.parentsc0

***Predicting probabilities for the estimation subsample**
predict pcens0 if e(sample)

***Calculating probability of being uncensored**
replace pcens0=1-pcens0

***Calculating cumulative probabilities**

sort id time
by id: replace pcens0=pcens0*pcens0[_n-1] if _n!=1

***Calculating weights (stabilised and unstabilised)**
gen cens_w=1/pcens
gen cens_sw=pcens0/pcens

***checking distribution of weights**
summarize cens_w, detail
summarize cens_sw, detail

***Creating total weight (product of exposure and censoring weight)**
gen w=ipt_w*cens_w
gen sw=ipt_sw*cens_sw

***Checking distribution of weights**
summarize w, detail
summarize sw, detail

/*Estimating parameters from pooled negative binomial regression or log-binomial generalized linear model (weighted by sw)*/
glm v_ freqsum18 imd_cum epds0 i.moved0 i.mumemploy0 i.eduparents0 ///
i.mummarried0 nochild0 i.ethnicity0 i.parentsc0 [pw=sw], cluster(id) family(nbinomial ml) link(log) eform

glm v_18freqallb imd_cum epds0 i.moved0 i.mumemploy0 i.eduparents0 ///
i.mummarried0 nochild0 i.ethnicity0 i.parentsc0 [pw=sw], cluster(id) family(binomial) link(log) eform

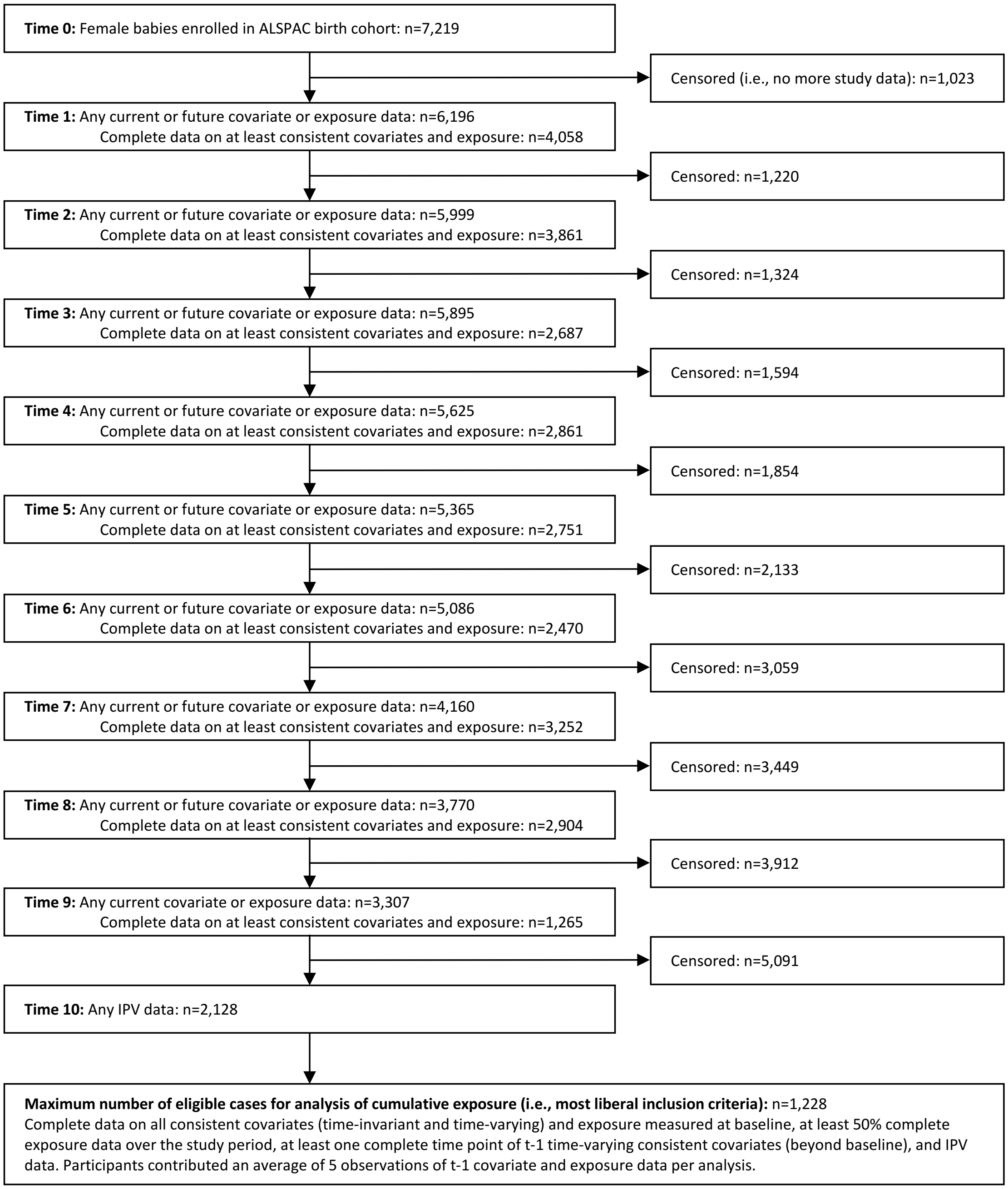

eFigure 2: **Flow chart illustrating study attrition (censoring and intermittent missingness) and the most liberal inclusion criteria used in analyses.** At each time t, uncensored participants are defined as those who have data at time t or time t+k, whereas censored participants are those who were permanently lost to follow-up after time t-1. Participants with complete data at time t (i.e., without any missing data on covariates or exposure at time t) are therefore a subset of the uncensored participants at time t. Selection bias due to non-random attrition was accounted for in multiple ways detailed in the main manuscript, including inverse probability weights for censoring (main analysis) as well as for intermittent missingness (sensitivity analysis).

eAppendix 3: Results

Longitudinal variation in exposure to neighbourhood-level deprivation over time

eFigure 3 shows the proportion of the sample living in the most deprived (IMD quintiles 4 and 5) versus the least deprived (IMD quintiles 1-3) neighbourhoods over the exposure period. Over time, the proportion of participants in the most deprived neighbourhoods decreased, likely influenced, at least in part, by non-random attrition.

eFigures 4-6 demonstrate the longitudinal variation in exposure to neighbourhood-level deprivation within participants. eFigure 4 compares participants' IMD quintiles at gestation (baseline) and age 18. It shows that within each IMD quintile at baseline, 31-51% of participants were in a different IMD quintile by age 18. eFigure 5 shows the absolute sum of changes in the level of exposure to neighbourhood-level deprivation (IMD quintile) experienced by participants over the study period: 46% of participants experienced any change in their IMD quintile over the study period. Finally, eFigure 6 summarises the net or directional change in participants IMD quintile over the study period: 26% of participants experienced a net decrease and 16% a net increase in the severity of their neighbourhood deprivation exposure.

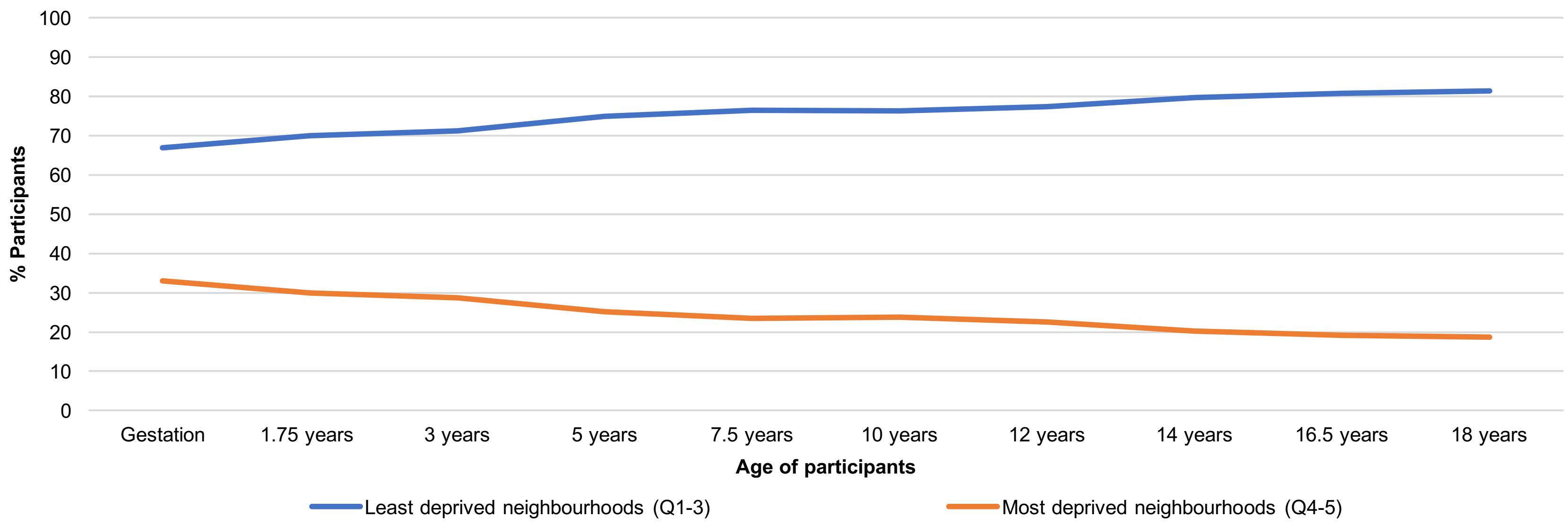

eFigure 3: **Percent of participants by exposure to most versus least deprived residential neighbourhoods during the period of exposure under study**

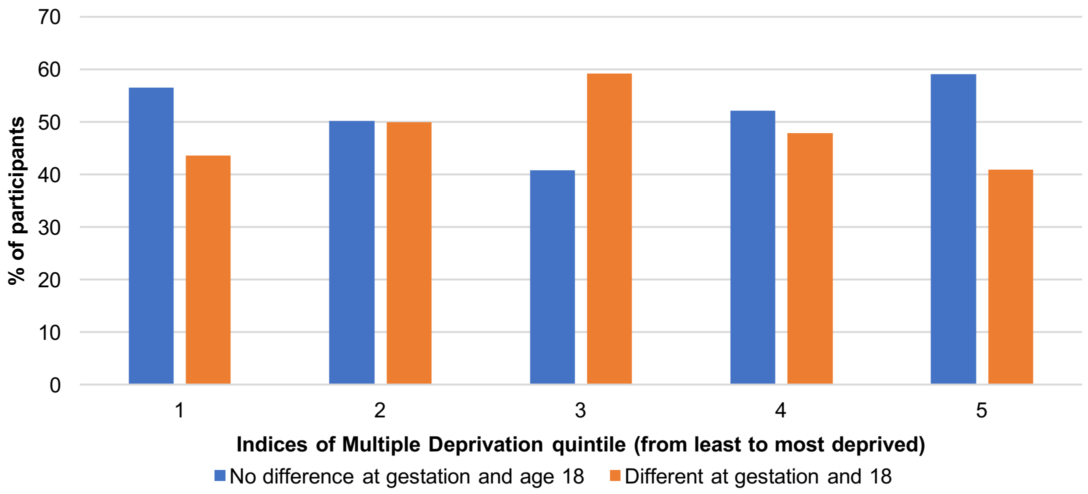

eFigure 4: **Percent of participants whose level of exposure to neighbourhood deprivation at gestation was different at age 18**. N=1,756 women with complete IMD data at gestation (baseline) and age 18.

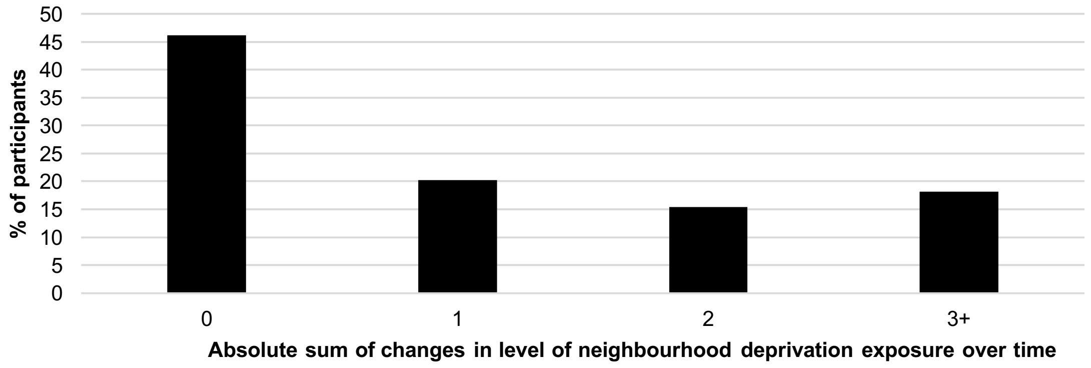

eFigure 5: **Percent of participants by absolute sum of changes in neighbourhood deprivation exposure experienced between ages 0-18**. N=1,692 women who were not missing data on neighbourhood deprivation exposure (IMD quintile) for more than 50% of time points between ages 0-18. For this figure, any missing data on neighbourhood deprivation exposure for these remaining participants were carried forward from the last available time point.

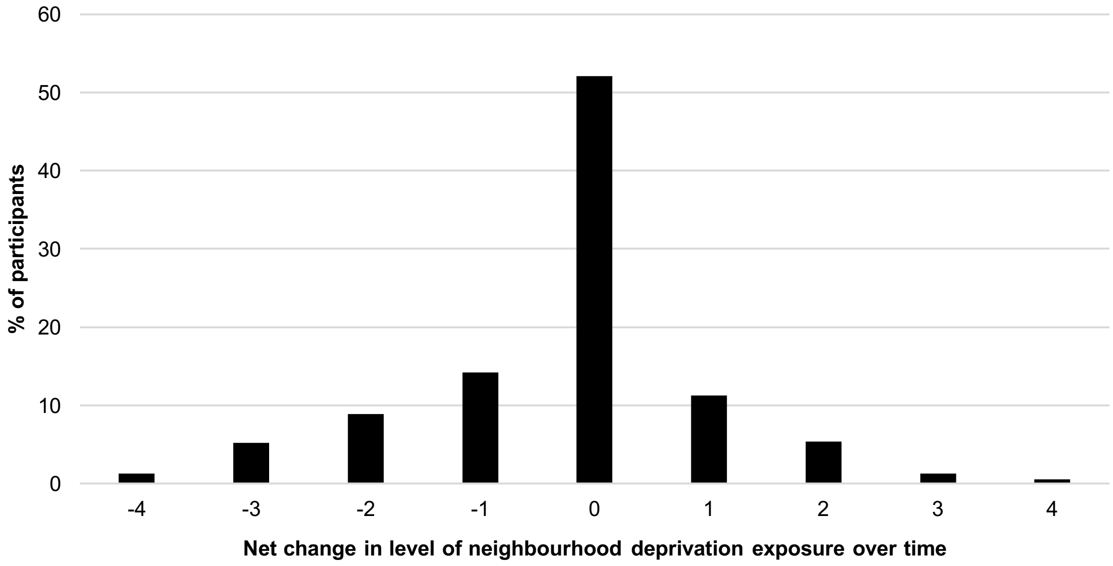

***eFigure 6:* Percent of participants by net change in level of neighbourhood deprivation exposure between ages 0-18**. N=1,692 women who were not missing data on neighbourhood deprivation exposure (IMD quintile) for more than 50% of time points between ages 0-18. For this figure, any missing data on neighbourhood deprivation exposure for these remaining participants were carried forward from the last available time point.

***eTable 3:* Prevalence of IPV victimisation and impact items and overall types between ages 18-21**

**Victimisation items**	**Experienced at least once (n=2,014): N (%)**
Told you who you could see, where you could go, or regularly checked what you were doing and where you were	346 (17)
Made fun of you, called you hurtful names, shouted at you	443 (22)
Used physical force such as pushing, slapping, hitting or holding you down	245 (12)
Used more severe physical force such as punching, strangling, beating you up, hitting you with an object	96 (5)
Pressured you into kissing/touching/something else	144 (7)
Physically forced you into kissing/touching/something else	72 (4)
Pressured you into having sexual intercourse	192 (10)
Physically forced you into having sexual intercourse	64 (3)
**Impact items**	**Experienced after IPV (n=588 who experienced any IPV): N (%)**
Scared	288 (49)
Upset/unhappy	465 (79)
Angry/annoyed	441 (75)
Made me feel sad	425 (72)
Affected my work/studies	187 (32)
Anxious	272 (46)
Depressed	231 (39)
No effect/not bothered	74 (74)

Made me drink more alcohol/take more drugs	92 (16)
Thought it was funny	48 (8)
Felt loved/protected/wanted	80 (14)
**IPV typologies**	**Any IPV (n= 2128): N (%)**
Any physical, psychological, or sexual IPV	683 (32)
Any physical, psychological, or sexual IPV with at least one of eight self-reported negative impacts	608 (29)
Any physical, psychological, or sexual IPV, excluding made fun of you, call you names, shouted	561 (26)
Any physical, sexual, or moderate-intensity (at least one item occurring at least a few times) psychological IPV	625 (29)
Any physical, sexual, or severe-intensity (at least one item occurring often) psychological IPV	443 (21)
Any physical or psychological IPV	617 (29)
Any sexual IPV	252 (12)

eTable 4: Distribution of IMD quintiles in categorical confounders at baseline

	**IMD quintiles: N (%)**				
	**1 (Least disadvantaged)**	**2**	**3**	**4**	**5 (Most disadvantaged)**
Lives with biological parents, N (%)					
Both parents	1 173 (96)	974 (92)	673 (91)	653 (88)	445 (79)
Either biological mother or father or neither	52 (4)	85 (8)	65 (9)	92 (12)	121 (21)
Mother recently moved house, N (%)					
Yes	148 (11)	124 (11)	86 (10)	106 (12)	93 (14)
No	1 187 (89)	1,041 (89)	763 (90)	765 (88)	574 (86)
Parental employment[2], N (%)					
Yes	841 (65)	765 (66)	531 (60)	544 (61)	329 (46)
No	454 (35)	387 (34)	349 (40)	342 (39)	384 (54)
Ethnicity					
Non-White	22 (2)	17 (2)	11 (2)	24 (5)	30 (9)
White	929 (98)	760 (98)	527 (98)	475 (95)	312 (91)
At least one parent has higher than O-level education					
Yes	959 (70)	764 (62)	479 (52)	418 (45)	254 (35)
No	420 (30)	462 (38)	444 (48)	503 (55)	482 (65)
At least one parent is part of lower social class					
Yes	172 (14)	201 (19)	163 (21)	225 (30)	250 (45)
No	1 022 (86)	851 (81)	602 (79)	523 (70)	303 (55)
Mother married					
Yes	1 224 (81)	993 (81)	734 (80)	667 (71)	414 (54)
No	164 (12)	239 (19)	189 (20)	273 (29)	355 (46)

eTable 5: Distribution of IMD quintiles in categorical confounders at Age 18

	**IMD quintiles: N (%)**				
	**1 (Least disadvantaged)**	**2**	**3**	**4**	**5 (Most disadvantaged)**
Lives with biological parents, N (%)					
Both parents	401 (78)	245 (75)	160 (70)	83 (60)	37 (61)
Either biological mother or father or neither	116 (22)	83 (25)	69 (30)	56 (40)	24 (39)
Mother recently moved house, N (%)					
Yes	19 (4)	11 (3)	11 (5)	3 (2)	4 (6)
No	500 (96)	319 (97)	217 (95)	138 (98)	60 (94)

Parental employment[2], N (%)					
Yes	365 (70)	229 (69)	154 (67)	77 (55)	34 (52)
No	156 (30)	104 (31)	76 (33)	64 (45)	31 (48)

Sensitivity and secondary analyses for the effect of neighbourhood deprivation on IPV among women

In addition to the secondary analyses to explore alternative hypotheses described in the main manuscript, we ran three types of sensitivity analyses (n=38 analyses) to test the robustness of our findings from our main analyses:

(1) ***Outcome operationalisation:*** We tested the robustness of our findings to several stricter definitions of IPV. These included: (a) 1=any IPV with at least one of the eight self-reported negative impacts versus 0=otherwise; (b) both (i) the average frequency and (ii) any experience of IPV, excluding emotional abuse (i.e., made fun of, insulted, shouted); and (c) based on a recent measurement study,[29] any physical, sexual, or (i) moderate-intensity (i.e., at least one item occurring at least a few times since age 18) or (ii) severe-intensity (i.e., at least one item occurring many times) psychological IPV.

(2) ***Additional missing data strategies***, including: (a) creating stabilised weights for intermittent missingness (so that final weights were the product of cumulative probabilities of exposure, censoring, and intermittent missingness histories);[28] (b) based on prior marginal structural model studies,[26] imputing missing covariate data with the last available observation (if t-1 missing, data set to missing), and (c) setting missing weights to 1, so that all complete participant-observation cases would be included (even when t-1 was missing), in both (i) long and (ii) wide data format to demonstrate consistency.

(3) ***Alternative model specifications***. This included (a) re-running analyses using time-varying, point-in-time exposure, similar to a prior marginal structural model study.[12] In the current study, this estimated the average effect of living in more versus less deprived neighbourhoods at each time point up until age 18 on IPV risk between ages 18-21. We also (b) used covariate and exposure data from the last available time period (T_6) to estimate the T_9 weights, to test the influence of using cross-sectional data in the main analyses, and (c) re-ran analyses using the available-covariate weights but excluding T_8, to test the influence of this time period (which was not included in analyses using consistent-covariate weights). Additionally, (d) we conducted conventional analyses in the un-weighted sample (i.e., not accounting for time-varying confounding) to compare methods. That is, in a crude model, we regressed each primary outcome onto exposure and all time-invariant and time-varying covariates measured at baseline and in an adjusted model we added the average value of time-varying covariates over the remaining time period.

Validity checks

The weight distributions for each relevant sensitivity and secondary analysis are shown in eTable 6.

eTable 6: **Mean, standard deviation, and range of stabilised weights by sensitivity analysis**

	M (SD)	1st percentile, 99th percentile
**Stabilized weights including weights for intermittent missingness**		
**Consistent-covariate exposure weights**	1.0 (0.1)	0.8, 1.2
**Consistent-covariate censoring weights**	0.9 (0.1)	0.7, 1.0
**Consistent-covariate intermittent missingness weights**	0.9 (0.1)	0.6, 1.0
**Consistent-covariate exposure*censoring*missing weights**	0.8 (0.1)	0.4, 1.0
**Available-covariate exposure weights**	1.0 (0.1)	0.7, 1.2
**Available-covariate censoring weights**	0.9 (0.1)	0.7, 1.0
**Available-covariate intermittent missingness weights**	0.8 (0.1)	0.4, 1.0
**Available-covariate exposure*censoring*missing weights**	0.7 (0.2)	0.3, 1.0
**Missing data on all available covariates imputed with last observation carried forward**		
**Available-covariate exposure weights**	1.0 (0.1)	0.8, 1.3
**Available-covariate censoring weights**	0.9 (0.1)	0.7, 1.1
**Available-covariate exposure*censoring weights**	0.9 (0.1)	0.6, 1.2
**Missing weights set to 1**		
**Consistent-covariate exposure weights**	1.0 (0.1)	0.5, 1.2
**Consistent-covariate censoring weights**	0.9 (0.1)	0.6, 1.0
**Consistent-covariate exposure*censoring weights**	0.9 (0.1)	0.4, 1.1
**Available-covariate exposure weights**	1.0 (0.2)	0.1, 1.2

	M (SD)	1st percentile, 99th percentile
**Available-covariate censoring weights**	0.8 (0.1)	0.6, 1.0
**Available-covariate exposure*censoring weights**	0.8 (0.2)	0.04, 1.1
**Main analysis but with T9 weights using covariate data from T6 (instead of T9)**		
**Consistent-covariate exposure weights**	1.0 (0.1)	0.8, 1.3
**Consistent-covariate censoring weights**	0.9 (0.04)	0.8, 1.0
**Consistent-covariate exposure*censoring weights**	0.9 (0.1)	0.7, 1.2
**Available-covariate exposure weights**	1.0 (0.1)	0.7, 1.3
**Available-covariate censoring weights**	0.9 (0.1)	0.7, 1.2
**Available-covariate exposure*censoring weights**	0.9 (0.1)	0.6, 1.2
**Main analysis with available-covariate weights but excluding T8**		
**Available-covariate exposure weights**	1.0 (0.1)	0.8, 1.2
**Available-covariate censoring weights**	0.9 (0.1)	0.7, 1.0
**Available-covariate exposure*censoring weights**	0.9 (0.1)	0.6, 1.1
**Crime deprivation (Quintile 4 and 5 vs. Quintiles 1, 2, and 3)**		
**Consistent-covariate exposure weights**	1.0 (0.1)	0.8, 1.2
**Consistent-covariate censoring weights**	0.9 (0.1)	0.7, 1.0
**Consistent-covariate exposure*censoring weights**	0.9 (0.1)	0.7, 1.1
**Available-covariate exposure weights**	1.0 (0.1)	0.8, 1.2
**Available-covariate censoring weights**	0.9 (0.1)	0.7, 1.0
**Available-covariate exposure*censoring weights**	0.9 (0.1)	0.6, 1.1
**Income deprivation (Quintile 4 and 5 vs. Quintiles 1, 2, and 3)**		
**Consistent-covariate exposure weights**	1.0 (0.1)	0.8, 1.3
**Consistent-covariate censoring weights**	0.9 (0.1)	0.7, 1.0
**Consistent-covariate exposure*censoring weights**	0.9 (0.1)	0.6, 1.2
**Available-covariate exposure weights**	1.0 (0.1)	0.8, 1.2
**Available-covariate censoring weights**	0.9 (0.1)	0.7, 1.0
**Available-covariate exposure*censoring weights**	0.9 (0.1)	0.6, 1.1
**Ordinal neighbourhood deprivation (quintile variable)**		
**Consistent-covariate exposure weights**	1.0 (0.1)	0.8, 1.1
**Consistent-covariate censoring weights**	0.9 (0.1)	0.7, 1.0
**Consistent-covariate exposure*censoring weights**	0.9 (0.1)	0.7, 1.1
**Available-covariate exposure weights**	1.0 (0.1)	0.6, 1.2
**Available-covariate censoring weights**	0.9 (0.1)	0.7, 1.0
**Available-covariate exposure*censoring weights**	0.9 (0.1)	0.5, 1.0

M is mean. SD is standard deviation.

Effect estimates

eTable 7 shows the effect estimates from all sensitivity analyses.

***eTable 7:* Sensitivity analyses for effect estimate of neighbourhood deprivation on IPV among women testing the robustness of the main findings**

	N_{women} ($N_{observations}$) in analysis	Relative risk	95% CI
**Outcome: Any IPV with a negative impact (binary)**			
**Consistent-covariate weights**	1,226 (6,279)	1.27	0.96, 1.69
**Available-covariate weights**	1,040 (5,067)	1.39	1.00, 1.93
**Outcome: IPV, excluding emotional abuse**			
**Outcome: IPV frequency score (count)**			
**Consistent-covariate weights**	1,228 (6,294)	1.74	1.05, 2.86
**Available-covariate weights**	1,041 (5,088)	1.68	1.04, 2.71
**Outcome: Any IPV (binary)**			
**Consistent-covariate weights**	1,228 (6,294)	1.42	1.05, 1.92
**Available-covariate weights**	1,041 (5,088)	1.37	0.97, 1.94
**Outcome: Any of physical, sexual, or moderate psychological IPV**			
**Consistent-covariate weights**	1,228 (6,294)	1.35	1.02, 1.78
**Available-covariate weights**	1,041 (5,088)	1.35	0.98, 1.86
**Outcome: Any of physical, sexual, or severe psychological IPV**			
**Consistent-covariate weights**	1,228 (6,294)	1.34	0.93, 1.93
**Available-covariate weights**	1,041 (5,088)	1.34	0.89, 2.03
**Stabilised weights including weights for intermittent missingness**			
**Outcome: IPV frequency score (count)**			
**Consistent-covariate weights**	1,228 (6,294)	1.61	1.11, 2.33
**Available-covariate weights**	1,041 (5,088)	1.72	1.10, 2.69
**Outcome: Any IPV (binary)**			
**Consistent-covariate weights**	1,228 (6,294)	1.38	1.07, 1.79
**Available-covariate weights**	1,041 (5,088)	1.39	1.04, 1.86
**Missing data on all available covariates imputed with last observation**			
**Outcome: IPV frequency score (count)**	1,041 (6,454)	1.69	1.09, 2.65

	N_{women} ($N_{observations}$) in analysis	Relative risk	95% CI
**Outcome: Any IPV (binary)**	1,041 (6,454)	1.37	1.01, 1.86
**Missing weights set to 1**			
**Outcome: IPV frequency score (count)**			
**Consistent-covariate weights**	1,228 (9,824)	1.61	1.14, 2.27
**Available-covariate weights**	1,041 (9,369)	1.68	1.13, 2.51
**Outcome: Any IPV (binary)**			
**Consistent-covariate weights**	1,228 (9,824)	1.35	1.07, 1.70
**Available-covariate weights**	1,041 (9,369)	1.42	1.08, 1.87
**Missing weights set to 1 in wide dataset (unit of analysis is participant)**			
**Outcome: IPV frequency score (count)**			
**Consistent-covariate weights**	1,228 (N/A)	1.59	1.11, 2.26
**Available-covariate weights**	1,041 (N/A)	1.78	1.20, 2.65
**Outcome: Any IPV (binary)**			
**Consistent-covariate weights**	1,228 (N/A)	1.31	1.03, 1.66
**Available-covariate weights**	1,041 (N/A)	1.46	1.11, 1.92
**Exposure: Point-in-time exposure (binary, time-varying)**			
**Outcome: IPV frequency score (count)**			
**Consistent-covariate weights**	1,291 (6,400)	1.44	1.11, 1.87
**Available-covariate weights**	1,112 (5,237)	1.40	1.01, 1.94
**Outcome: Any IPV (binary)**			
**Consistent-covariate weights**	1,291 (6,400)	1.29	1.06, 1.57
**Available-covariate weights**	1,112 (5,237)	1.23	0.99, 1.55
**T9 weights using covariate data from T6 (instead of T9)**			
**Outcome: IPV frequency score (count)**			
**Consistent-covariate weights**	1,228 (6,340)	1.64	1.12, 2.41
**Available-covariate weights**	1,041 (5,180)	1.65	1.03, 2.63
**Outcome: Any IPV (binary)**			
**Consistent-covariate weights**	1,228 (6,340)	1.40	1.08, 1.82
**Available-covariate weights**	1,041 (5,180)	1.37	1.01, 1.86
**Main analysis with available-covariate weights but excluding T8**			
**Outcome: IPV frequency score (count)**	1,066 (4,862)	1.62	1.04, 2.52
**Outcome: Any IPV (binary)**	1,066 (4,862)	1.36	1.01, 1.83
**Un-weighted (conventional) analyses of cumulative exposure**			
**Outcome: IPV frequency score (count)**			
**Crude analysis (adjusting for only baseline covariates)[a]**	1,123 (N/A)	1.63	1.12, 2.35
**Adjusted analysis (for baseline and time-varying covariates)[b]**	1,021 (N/A)	1.56	1.04, 2.34
**Outcome: Any IPV (binary)**			
**Crude analysis (adjusting for only baseline covariates)[a]**	1,123 (N/A)	1.44	1.11, 1.86
**Adjusted analysis (for baseline and time-varying covariates)[b]**	1,021 (N/A)	1.44	1.09, 1.91

N is number. CI is confidence interval. MSM is marginal structural model. N/A is not applicable.
Unless otherwise noted, analysis is negative binomial regression (for count outcome) or log-binomial generalised linear model (for binary outcome), weighted as indicated, conducted in a long-form data set (with participant-time as the unit of analysis) with clustering accounted for with robust (conservative) standard errors and adjusting for baseline time-invariant and time-varying covariates. Relative risk in the log negative binomial regression is the incidence rate ratio and in the log-binomial model is the risk ratio.
[a]Conventional generalised linear model adjusting for all available covariates at baseline in unweighted sample in wide format.
[b]Conventional generalised linear model adjusting for all available covariates at baseline and the average value of all available time-varying covariates in unweighted sample in wide format.

eTable 8 shows the effect estimates from all secondary analyses exploring alternative hypotheses. As we expected given the exponential distribution of the deprivation scores, we did not find a meaningful association between the ordinal exposure variable and risk of IPV. To confirm the robustness of these findings, we also re-ran our analyses using exposure weights constructed by multinomial rather than ordinal logistic regression (in case of violations of the proportional odds assumption). However, results did not differ and are thus not shown.

***eTable 8:* Secondary analyses for effect estimate of neighbourhood deprivation on IPV among women exploring alternative hypotheses**

	N_{women} ($N_{observations}$) in analysis	Relative risk	95% CI
**Crime deprivation**			
**Outcome: IPV frequency score (count)**			
**Consistent-covariate weights**	1,185 (6,196)	1.18	0.87, 1.62
**Available-covariate weights**	1,041 (5,088)	1.25	0.89, 1.76
**Outcome: Any IPV (binary)**			
**Consistent-covariate weights**	1,185 (6,196)	1.12	0.89, 1.41
**Available-covariate weights**	1,041 (5,088)	1.16	0.90, 1.50
**Income deprivation**			
**Outcome: IPV frequency score (count)**			

	N_{women} ($N_{observations}$) in analysis	Relative risk	95% CI
Consistent-covariate weights	1,185 (6,196)	1.20	0.88, 1.64
Available-covariate weights	1,041 (5,088)	0.99	0.63, 1.57
Outcome: Any IPV (binary)			
Consistent-covariate weights	1,185 (6,196)	1.13	0.85, 1.51
Available-covariate weights	1,041 (5,088)	1.00	0.71, 1.37
**Ordinal neighbourhood deprivation**			
Outcome: IPV frequency score (count)			
Consistent-covariate weights	1,185 (6,196)	1.04	0.92, 1.18
Available-covariate weights	1,041 (5,088)	1.01	0.87, 1.16
Outcome: Any IPV (binary)			
Consistent-covariate weights	1,185 (6,196)	1.07	0.97, 1.18
Available-covariate weights	1,041 (5,088)	1.06	0.94, 1.18
**Physical or psychological IPV**			
Outcome: IPV frequency score (count)			
Consistent-covariate weights	1,228 (6,294)	1.54	1.06, 2.24
Available-covariate weights	1,041 (5,088)	1.49	0.95, 2.33
Outcome: Any IPV (binary)			
Consistent-covariate weights	1,228 (6,294)	1.51	1.15, 1.99
Available-covariate weights	1,041 (5,088)	1.41	1.02, 1.95
**Sexual IPV**			
Outcome: IPV frequency score (count)			
Consistent-covariate weights	1,228 (6,294)	1.86	0.99, 3.49
Available-covariate weights	1,041 (5,088)	2.28	1.13, 4.60
Outcome: Any IPV (binary)			
Consistent-covariate weights	1,228 (6,294)	1.33	0.81, 2.21
Available-covariate weights	1,041 (5,088)	1.59	0.88, 2.87
**No exposure versus past versus most recent (ordinal)[a]**			
Outcome: IPV frequency score (count)			
Consistent-covariate weights	1,101 (5,786)		
Past exposure to deprived neighbourhood		1.70	1.22, 2.35
Living in deprived neighbourhood at age 18		1.25	0.82, 1.91
Available-covariate weights	951 (4,714)		
Past exposure to deprived neighbourhood		1.39	0.95, 2.01
Living in deprived neighbourhood at age 18		1.18	0.72, 1.92
Outcome: Any IPV (binary)			
Consistent-covariate weights	1,101 (5,786)		
Past exposure to deprived neighbourhood		1.64	1.25, 2.14
Living in deprived neighbourhood at age 18		1.21	0.88, 1.66
Available-covariate weights	951 (4,714)		
Past exposure to deprived neighbourhood		1.55	1.14, 2.10
Living in deprived neighbourhood at age 18		1.10	0.76, 1.60

N is number. CI is confidence interval. MSM is marginal structural model. Unless otherwise noted, analysis is negative binomial regression (for count outcome) or log-binomial generalised linear model (for binary outcome), weighted as indicated, conducted in a long-form data set (with participant-time as the unit of analysis) with clustering accounted for with robust (conservative) standard errors and adjusting for baseline time-invariant and time-varying covariates. Relative risk in the negative binomial regression is the incidence rate ratio and in the log-binomial model is the risk ratio.

[a]Referent is never lived in most deprived neighbourhoods. Only n=2 participants were living in a deprived neighbourhood at age 18 without ever previously living in a deprived neighbourhood. As such, these participants were analysed in the same category as all other participants living in a deprived neighbourhood at age 18.

References

1 Benson ML, Fox GL, DeMaris A, van Wyk JA. Neighborhood Disadvantage, Individual Economic Distress and Violence Against Women in Intimate Relationships. Journal of Quantitative Criminology. 2003;19:207-35.

2 Giordano PC, Copp JE, Longmore MA, Manning WD. Anger, Control, and Intimate Partner Violence in Young Adulthood. Journal of Family Violence. 2016;31(1):1-13.

3 Gomez AM. Testing the Cycle of Violence Hypothesis: Child Abuse and Adolescent Dating Violence as Predictors of Intimate Partner Violence in Young Adulthood. Youth & Society. 2011;43(1):171-92.

4 Jain S, Buka Stephen L, Subramanian SV, Molnar Beth E. Neighborhood predictors of dating violence victimization and perpetration in young adulthood: A multilevel study US: US; 2010 [updated 2010. 9:[1737-44]. Available from: http://ovidsp.ovid.com/ovidweb.cgi?T=JS&PAGE=reference&D=psyc7&NEWS=N&AN=2010-25205-019
http://www.ncbi.nlm.nih.gov/pmc/articles/PMC2920975/pdf/1737.pdf.

5 Leddy AM, Lippman SA, Neilands TB, Twine R, Ahern J, Gomez-Olive FX, et al. Community collective efficacy is associated with reduced physical intimate partner violence (IPV) incidence in the rural province of Mpumalanga, South Africa: findings from HPTN 068. J Epidemiol Community Health. 2018.
6 Yakubovich AR, Stöckl H, Murray J, Melendez-Torres GJ, Steinert JI, Glavin CEY, et al. Risk and protective factors for intimate partner violence against women: systematic review and meta-analyses of prospective–longitudinal studies. American Journal of Public Health. 2018;108(7):e1-e11.
7 van Wyk JA, Benson ML, Fox GL, DeMaris A. Detangling Individual-, Partner-, and Community-Level Correlates of Partner Violence. Crime & Delinquency. 2003;49(3):412-38.
8 Fox GL, Benson ML, DeMaris AA, van Wyk J. Economic distress and intimate violence: Testing family stress and resources theories. Journal of Marriage and the Family, Living, Marriage and Family Living. 2002;64:793-807.
9 Benson ML, Wooldredge J, Thistlethwaite AB, Fox GL. The Correlation between Race and Domestic Violence is Confounded with Community Context. Social Problems. 2004;51(3):326-42.
10 DeMaris A, Benson ML, Fox GL, Hill TD, Wyk JV. Distal and proximal factors in domestic violence: A test of an integrated model. Journal of Marriage and Family. 2003;65:652-67.
11 Capaldi DM, Knoble NB, Shortt JW, Kim HK. A systematic review of risk factors for intimate partner violence. Partner abuse. 2012;3(2):231-80.
12 Cerda M, Diez-Roux AV, Tchetgen ET, Gordon-Larsen P, Kiefe C. The relationship between neighborhood poverty and alcohol use: estimation by marginal structural models. Epidemiology. 2010;21(4):482-9.
13 Wodtke GT. Duration and timing of exposure to neighborhood poverty and the risk of adolescent parenthood. Demography. 2013;50:1765-88.
14 Wodtke GT, Harding DJ, Elwert F. Neighborhood Effects in Temporal Perspective. Am Sociol Rev. 2011;76(5):713-36.
15 Textor J, van der Zander B, Gilthorpe MK, Liskiewicz M, Ellison GTH. Robust causal inference using directed acyclic graphs: the R package 'dagitty'. Int J Epidemiol. 2016;45(6):1887-94.
16 Daniel RM, Cousens SN, De Stavola BL, Kenward MG, Sterne JA. Methods for dealing with time-dependent confounding. Stat Med. 2013;32(9):1584-618.
17 Hernan MA, Robins JM. Estimating causal effects from epidemiological data. J Epidemiol Community Health. 2006;60(7):578-86.
18 Robins JM, Hernán MA, Brumback B. Marginal structural models and causal inference in epidemiology. Epidemiology. 2000;11(5):550-60.
19 Hernán MA, Brumback B, Robins JM. Marginal structural models to estimate the causal effect of Zidovudine on the survival of HIV-positive men. Epidemiology. 2000(11):5.
20 Lad M. The English Indices of Multiple Deprivation 2010. London: Department for Communities and Local Government; 2011.
21 Gill B. The English Indices of Deprivation 2015. England: Department for Communities and Local Government; 2015.
22 McLennan D, Barnes H, Noble M, Davies J, Garratt E. The English Indices of Deprivation 2010. London: Department for Communities and Local Government; 2011.
23 Smith T, Noble M, Noble S, Wright G, McLennan D, Plunkett E. The English Indices of Deprivation 2015: Technical report. London: Department for Communities and Local Government; 2015.
24 Cox JL, Holden JM, Sagovsky R. Detection of postnatal depression: Development of the 10-item Edinburgh Postnatal Depression Scale. British Journal of Psychiatry. 1987;150:782-6.
25 Cole SR, Hernan MA. Constructing inverse probability weights for marginal structural models. Am J Epidemiol. 2008;168(6):656-64.
26 Fewell Z, Hernan MA, Wolfe F, Tilling K, Choi H, Sterne JAC. Controlling for time-dependent confounding using marginal structural models. The Stata Journal. 2004;4(4):402-20.
27 Sharkey P, Elwert F. The legacy of disadvantage: Multigenerational neighborhood effects on cognitive ability. American Journal of Sociology. 2011;116(6):1934-81.
28 VanderWeele TJ, Hawkley LC, Thisted RA, Cacioppo JT. A marginal structural model analysis for loneliness: implications for intervention trials and clinical practice. J Consult Clin Psychol. 2011;79(2):225-35.

29 Heise L, Pallitto C, García-Moreno C, Clark CJ. Measuring psychological abuse by intimate partners: Constructing a cross-cultural indicator for the Sustainable Development Goals. SSM - Population Health. 2019:100377.
